# Changes in anticoagulant prescription patterns over time for patients with atrial fibrillation around the world

**DOI:** 10.1002/joa3.12588

**Published:** 2021-07-10

**Authors:** Monika Kozieł, Christine Teutsch, Valentina Bayer, Shihai Lu, Venkatesh K. Gurusamy, Jonathan L. Halperin, Kenneth J. Rothman, Hans‐Christoph Diener, Chang‐Sheng Ma, Menno V. Huisman, Gregory Y. H. Lip, the GLORIA‐AF Investigators

**Affiliations:** ^1^ Liverpool Centre for Cardiovascular Science University of Liverpool and Liverpool Heart and Chest Hospital Liverpool UK; ^2^ Department of Cardiology and Angiology Silesian Centre for Heart Diseases Zabrze Poland; ^3^ Department of Clinical Development and Medical Affairs Therapeutic Area Cardiometabolism Boehringer Ingelheim International GmbH Ingelheim Germany; ^4^ Biostatistics and Data Sciences Department Boehringer Ingelheim Pharmaceuticals, Inc. Ridgefield CT USA; ^5^ Global Epidemiology Boehringer Ingelheim International GmbH Ingelheim Germany; ^6^ Icahn School of Medicine at Mount Sinai New York City NY USA; ^7^ RTI Health Solutions Research Triangle Park NC USA; ^8^ Institute for Medical Informatics, Biometry and Epidemiology Essen Germany; ^9^ University of Duisburg‐Essen Essen Germany; ^10^ Cardiology Department Atrial Fibrillation Center Beijing Anzhen Hospital Capital Medical University Beijing China; ^11^ Department of Thrombosis and Hemostasis Leiden University Medical Center Leiden the Netherlands; ^12^ Aalborg Thrombosis Research Unit Department of Clinical Medicine Aalborg University Aalborg Denmark

**Keywords:** atrial fibrillation, bleeding risk, GLORIA‐AF, oral anticoagulants, stroke risk

## Abstract

**Background:**

Prescribing patterns for stroke prevention in atrial fibrillation (AF) patients evolved with approval of non‐Vitamin K antagonist oral anticoagulants (NOACs) over time.

**Objectives:**

To assess changes in anticoagulant prescription patterns in various geographical regions upon first approval of a NOAC and to analyze the evolution of oral anticoagulants (OACs) use over time in relation to CHA_2_DS_2_‐VASc and HAS‐BLED risk profiles.

**Methods:**

Global Registry on Long‐Term Oral Antithrombotic Treatment in Patients with Atrial Fibrillation **(**GLORIA‐AF) Phases II and III reported data on antithrombotic therapy for patients with newly diagnosed AF and ≥1 stroke risk factor. We focused on sites enrolling patients in both phases and reported treatment patterns for the first 4 years after initial NOAC approval.

**Results:**

From GLORIA‐AF Phases II and III, 27 432 patients were eligible for this analysis. When contrasting the first year with the fourth year of enrolment, the proportion of NOAC prescriptions increased in Asia from 29.2% to 60.8%, in Europe from 53.4% to 75.8%, in North America from 49.0% to 73.9% and in Latin America from 55.7% to 71.1%. The proportion of Vitamin K antagonists (VKAs) use decreased across all regions over time, in Asia from 26.0% to 9.8%, in Europe from 35.5% to 16.8%, in North America from 28.9% to 12.1%, and in Latin America from 32.4% to 17.8%. In the multivariable analysis, factors associated with NOAC prescription were as follows: enrolment year, type of site, region, stroke and bleeding risk scores, and type and categorization of AF.

**Conclusions:**

During 4 years after the approval of the first NOAC, NOAC use increased, while VKA use decreased, across all regions.

## Graphical abstract

Treatment patterns for stroke prevention in AF overtime were assessed globally. The novelty of this study refers to evaluation of changes upon first approval of a NOAC. NOAC use increased and VKA use decreased over 4 years.

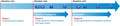

## 1 INTRODUCTION

Atrial fibrillation (AF) is the most common cardiac arrhythmia, with both incidence and prevalence increasing with age. Nonvalvular AF is associated with a fivefold increase in risk of stroke. Therefore, stroke prevention is the cornerstone of the holistic approach to AF management. Currently, when oral anticoagulation (OAC) is indicated for stroke prevention in patients with AF, non‐Vitamin K antagonist oral anticoagulants (NOACs) are recommended in preference to Vitamin K antagonists (VKAs).^1^ In contrast, when NOACs were introduced and adopted into practice, clinical guidelines were still assessing results from pivotal trials. Since then, the rationale for using NOACs has changed. It is challenging to measure temporal trends of global prescription patterns, however, because the timing of NOAC approval varied across countries and prescription patterns can change rapidly as uptake of a new agent increases. Moreover, use of a particular OAC may reflect the manner in which physicians interpret stroke and bleeding risk scores, which have also been incorporated variably into clinical practice guidelines. Published descriptions of global NOAC uptake have not consistently accounted for these variables, overlooking distinctions based on the local availability of NOACs for clinical use. Therefore, results of such analyses are affected by the distribution of countries included in the evaluation.

The specific design of the large, prospective, global registry Global Registry on Long‐Term Oral Antithrombotic Treatment in Patients with Atrial Fibrillation (GLORIA‐AF) Phases II and III provides an opportunity to assess temporal changes over time on a global scale. Enrolment for Phases II and III continued from 2011 to 2016 allowing for assessment of changes in practice patterns in a large number of patients over more than 4 years.

This report is based on baseline data, including antithrombotic prescriptions for stroke prevention in patients with newly diagnosed AF enrolled in Phases II and III of GLORIA‐AF. We assessed temporal changes in antithrombotic prescription patterns within specific geographical regions, starting from initial NOAC approval. We also analyzed changes in types of OAC prescribed in relation to CHA_2_DS_2_‐VASc (heart failure, hypertension, age ≥75 years, diabetes, stroke/transient ischemic attack, vascular disease, age 65‐74 years, sex category) and HAS‐BLED (hypertension, abnormal renal/liver function, stroke, bleeding history or predisposition, labile international normalized ratio, elderly [>65 years], drugs or alcohol concomitantly) risk scores during each year of enrolment.

## 2 METHODS

The design of GLORIA‐AF (https://clinicaltrials.gov/ct2/home; trial registration numbers NCT01468701, NCT01671007, and NCT01937377) has been previously reported.^2^ A signed patient informed consent form was obtained before enrolment. The study protocol is concordant with the ethical guidelines of the 1975 Declaration of Helsinki.

**FIGURE 1:**
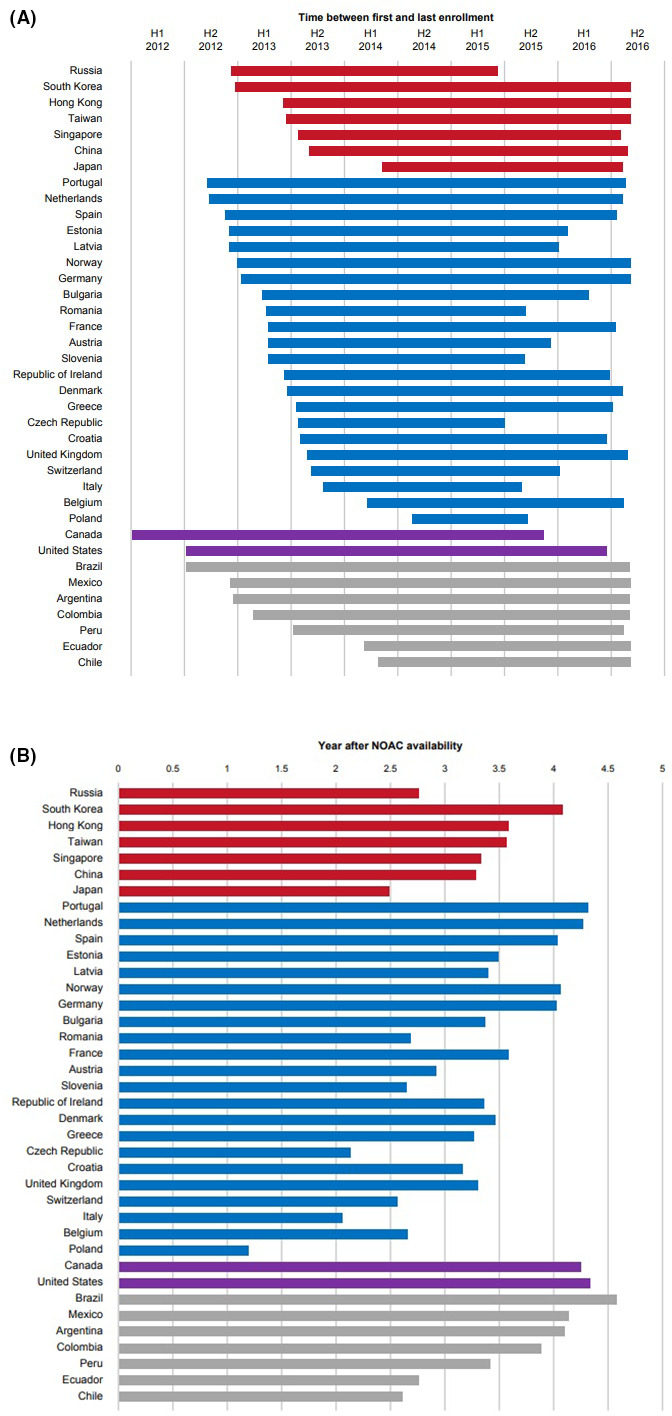
(A) Enrolment period in Phases II and III of the GLORIA‐AF registry per country in calendar dates. **(**B) Enrolment period in Phases II and III of the GLORIA‐AF registry per country in years after NOAC availability

The GLORIA‐AF study collected data in 44 countries regarding routine clinical practice for patients with AF diagnosed <3 months before the baseline visit (4.5 months in Latin America), to evaluate characteristics that influenced choice of antithrombotic treatment and clinical outcomes. GLORIA‐AF was conducted in three phases (Figure 1A,B). Phase I was before NOACs became available for stroke prevention in AF. In Phase I, a cross‐sectional approach was implemented. Phase II began when dabigatran etexilate (dabigatran) was approved in each participating country. During Phase II, baseline characteristics of all enrolled patients were collected, and those prescribed dabigatran were followed for 2 years. Phase III began once relevant baseline characteristics of patients initiating dabigatran and VKA in Phase II showed substantial overlap, as measured by comparison of propensity score distributions. During Phase III, follow‐up data were collected for up to 3 years regardless of prescribed antithrombotic therapy.^2^

Adults with nonvalvular AF and ≥1 CHA_2_DS_2_‐VASc risk factor score for stroke were included. Stroke and bleeding risks were assessed using the CHA_2_DS_2_‐VASc and HAS‐BLED risk scores.^3^, ^4^ Patients were managed according to local practice. This report includes regions and sites enrolling patients during Phases II and III.

Standard electronic case reports forms (eCRFs) were used to collect patients' baseline characteristics and follow‐up observation data. Baseline therapy was the treatment prescribed for long‐term anticoagulation subsequent to the diagnosis of AF and recorded at the baseline visit.

Time zero in a participating country was set to the date of the baseline visit for the first patient in each country. The first year of enrolment for a participating country was the first year after time zero in that country. Most countries continued enrolment for up to 4 years. In this paper, we classify newly enrolled patients according to which prescribed treatment they received at their baseline visit, by year of enrolment.

### 2.1 Statistical analysis

Treatment patterns are presented as a percentage of patients prescribed NOAC, VKA, or no OAC in each of the 4 years of enrolment, overall and by region. Categorical variables are reported as absolute frequencies and percentages, and continuous variables are summarized by median (Q1, Q3). Baseline characteristics were described by categorization of patients with AF according to stroke prevention treatment (NOAC, VKA, no OAC) and year of enrolment (first year versus last year, ie, Year 4), as well as by CHA_2_DS_2_‐VASc and HAS‐BLED risk scores. For each treatment, standardized differences were included to compare baseline characteristics between the last year and first year of enrolment.^5^

Factors associated with OAC prescription patterns over time were evaluated using log‐binomial regression models, providing estimates of relative probability for NOAC prescription (vs. VKA prescription). Both univariate and multivariable log‐binomial regression analyses were fit to evaluate the crude as well as adjusted probability ratios together with 95% confidence intervals (CIs).

Missing data were imputed using multiple imputation. The imputation model was constructed with 56 baseline patient characteristic variables including those used in the multivariable analyses. The COPY method was used to obtain approximate maximum likelihood estimates when log‐binomial models failed to converge.^6^ Statistical analyses were performed using SAS version 9.4 (SAS Institute, Inc, Cary, NC).

## 3 RESULTS

**FIGURE 2:**
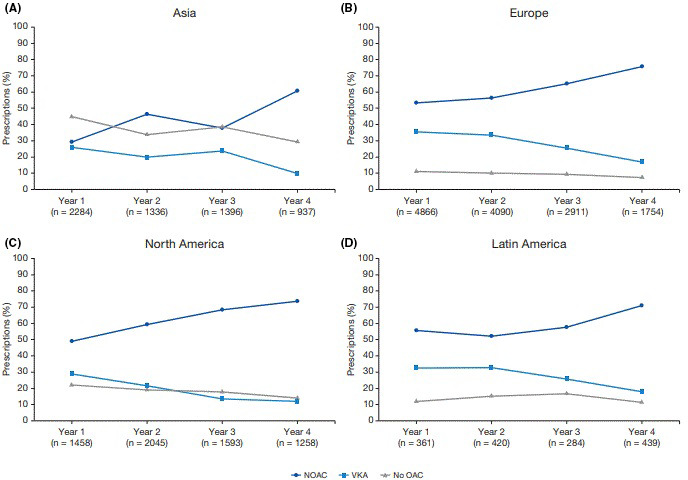
Temporal trends of antithrombotic therapy prescription globally. NOAC, non‐Vitamin K antagonist oral anticoagulants; OAC, oral anticoagulation; VKA, Vitamin K antagonists

**FIGURE 3:**
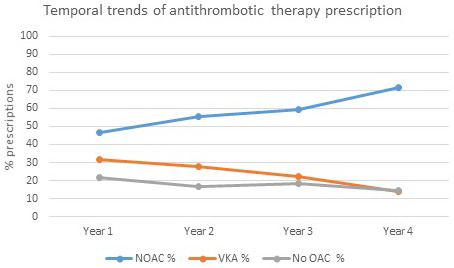
Temporal trends of antithrombotic therapy prescription by region. NOAC, non‐Vitamin K antagonist oral anticoagulants; OAC, oral anticoagulation; VKA, Vitamin K antagonists

There were 27 432 eligible patients who enrolled in GLORIA‐AF during Phases II and III and who qualified to be included in this analysis. Of 8969 patients who enrolled in the first year, 46.6% were prescribed NOAC, 31.9% were prescribed VKA, and 21.5% were prescribed no OAC. Of 4388 patients enrolled in the fourth year, 71.6% received NOAC, 14.1% received VKA, and 14.3% received no OAC (Figure 2). A similar trend in treatment pattern over time, ie, increase in NOAC and decrease in VKA, was observed for Europe and North America. From the third to fourth year, an increase in NOAC prescriptions and a decrease in VKA or no OAC prescription was reported in Asia (Figure 3). The prevalence of non‐OAC slightly decreased from Years 1‐4, except Latin America (Figure 2).

Baseline characteristics of patients prescribed NOAC by region are summarized in Table 1. Paroxysmal AF was less prevalent in patients with NOAC during the first year of enrolment than in patients with NOAC during the last year of enrolment in Europe, North America, and Latin America. The standardized differences for stroke and bleeding risk scores (CHA_2_DS_2_‐VASc and HAS‐BLED) between the last and first year for NOAC patients were small in Europe, North America, and Latin America (less than 0.1).

**TABLE 1:** Baseline Characteristics for NOAC patients by first and last year of enrollment by region

Characteristic	Asia			Europe			North America			Latin America		
	NOAC during first year (n = 666)	NOAC during last year (n = 570)	Std diff	NOAC during first year (n = 2598)	NOAC during last year (n = 1330)	Std diff	NOAC during first year (n = 714)	NOAC during last year (n = 930)	Std diff	NOAC during first year (n = 201)	NOAC during last year (n = 312)	Std diff
Age, median, IQR, y	71.0 (64.0‐78.0)	69.0 (63.0‐76.0)	−0.161	72.0 (65.0‐79.0)	73.0 (66.0‐79.0)	0.064	72.0 (65.0‐79.0)	71.0 (64.0‐77.0)	−0.121	73.0 (64.0‐79.0)	72.0 (65.0‐78.0)	−0.046
Females, n (%)	285 (42.8)	274 (48.1)	0.106	1191 (45.8)	611 (45.9)	0.002	305 (42.7)	429 (46.1)	0.069	93 (46.3)	164 (52.6)	0.126
Alcohol abuse, n (%)^a^	61 (9.2)	12 (2.1)	−0.310	185 (7.1)	65 (4.9)	−0.094	55 (7.7)	75 (8.1)	0.013	0 (0.0)	4 (1.3)	0.119
Unknown	24 (3.6)	60 (10.5)	0.273	277 (10.7)	125 (9.4)	−0.042	36 (5.0)	35 (3.8)	−0.062	3 (1.5)	6 (1.9)	0.033
BMI, median, IQR, kg/m^2^	25.1 (22.2‐28.4)	24.8 (22.6‐27.1)	−0.207	27.7 (24.9‐31.1)	27.9 (25.0‐31.5)	0.078	29.6 (25.7‐34.8)	30.2 (26.7‐35.5)	0.121	27.4 (24.6‐31.1)	27.4 (24.6‐31.2)	0.067
Missed, n (%)	26 (3.9)	14 (2.5)		21 (0.8)	2 (0.2)		0 (0.0)	2 (0.2)		0 (0.0)	0 (0.0)	
Type of AF, n (%)												
Paroxysmal	393 (59.0)	341 (59.8)	0.017	1212 (46.7)	708 (53.2)	0.132	422 (59.1)	645 (69.4)	0.215	83 (41.3)	158 (50.6)	0.188
Persistent	196 (29.4)	208 (36.5)	0.151	1028 (39.6)	492 (37.0)	−0.053	253 (35.4)	257 (27.6)	−0.169	70 (34.8)	73 (23.4)	−0.254
Permanent	77 (11.6)	21 (3.7)	−0.300	358 (13.8)	130 (9.8)	−0.125	39 (5.5)	28 (3.0)	−0.122	48 (23.9)	81 (26.0)	0.048
Categorization of AF, n (%)												
Symptomatic	218 (32.7)	156 (27.4)	−0.117	814 (31.3)	537 (40.4)	0.189	152 (21.3)	226 (24.3)	0.072	52 (25.9)	87 (27.9)	0.045
Minimally symptomatic	256 (38.4)	225 (39.5)	0.021	1030 (39.6)	411 (30.9)	−0.184	269 (37.7)	311 (33.4)	−0.089	81 (40.3)	86 (27.6)	−0.271
Asymptomatic	192 (28.8)	189 (33.2)	0.094	754 (29.0)	382 (28.7)	−0.007	293 (41.0)	393 (42.3)	0.025	68 (33.8)	139 (44.6)	0.221
Creatinine clearance (measured), median, IQR, ml/min	65.4 (50.8‐84.4)	68.9 (54.7‐87.4)	0.062	74.4 (56.5‐96.8)	76.5 (59.0‐99.4)	0.079	80.1 (59.7‐107.4)	83.8 (62.0‐112.7)	0.075	70.4 (54.5‐95.0)	67.3 (52.0‐87.1)	−0.038
<15, n (%)	0 (0.0)	1 (0.2)	0.028	15 (0.6)	3 (0.2)	−0.056	3 (0.4)	4 (0.4)	0.002	1 (0.5)	1 (0.3)	−0.028
15‐29, n (%)	13 (2.0)	8 (1.4)	−0.043	18 (0.7)	16 (1.2)	0.053	9 (1.3)	9 (1.0)	−0.028	5 (2.5)	3 (1.0)	−0.117
30‐49, n (%)	117 (17.6)	80 (14.0)	−0.097	319 (12.3)	154 (11.6)	−0.022	72 (10.1)	81 (8.7)	−0.047	16 (8.0)	33 (10.6)	0.090
50‐79, n (%)	248 (37.2)	198 (34.7)	−0.052	843 (32.4)	461 (34.7)	0.047	179 (25.1)	246 (26.5)	0.032	45 (22.4)	78 (25.0)	0.062
≥80, n (%)	169 (25.4)	142 (24.9)	−0.011	898 (34.6)	523 (39.3)	0.099	265 (37.1)	413 (44.4)	0.149	43 (21.4)	52 (16.7)	−0.121
Missing, n (%)	119 (17.9)	141 (24.7)	0.168	505 (19.4)	173 (13.0)	−0.175	186 (26.1)	177 (19.0)	−0.169	91 (45.3)	145 (46.5)	0.024
CHA_2_DS_2_‐VASc score, median, IQR	3.0 (2.0‐4.0)	3.0 (2.0‐3.0)	−0.271	3.0 (2.0‐4.0)	3.0 (2.0‐4.0)	−0.007	3.0 (2.0‐4.0)	3.0 (2.0‐4.0)	−0.003	3.0 (2.0‐4.0)	3.0 (2.0‐4.0)	0.020
HAS‐BLED score, median, IQR	1.0 (1.0‐2.0)	1.0 (0.0‐1.0)	−0.334	1.0 (1.0‐2.0)	1.0 (1.0‐2.0)	−0.028	1.0 (1.0‐2.0)	1.0 (1.0‐2.0)	−0.029	1.0 (1.0‐2.0)	1.0 (1.0‐2.0)	−0.086
Missing (HAS‐BLED), n (%)	50 (7.5)	80 (14.0)	0.212	354 (13.6)	173 (13.0)	−0.018	64 (9.0)	49 (5.3)	−0.144	23 (11.4)	6 (1.9)	−0.389
Medical history, n (%)												
Congestive heart failure	189 (28.4)	66 (11.6)	−0.430	669 (25.8)	265 (19.9)	−0.139	119 (16.7)	174 (18.7)	0.054	54 (26.9)	70 (22.4)	−0.103
Unknown	7 (1.1)	0 (0.0)	−0.128	23 (0.9)	13 (1.0)	0.010	6 (0.8)	1 (0.1)	−0.107	1 (0.5)	0 (0.0)	−0.059
Hypertension	523 (78.5)	411 (72.1)	−0.149	1965 (75.6)	1032 (77.6)	0.046	580 (81.2)	770 (82.8)	0.041	159 (79.1)	244 (78.2)	−0.022
Unknown	5 (0.8)	0 (0.0)	−0.103	6 (0.2)	4 (0.3)	0.014	1 (0.1)	0 (0.0)	−0.028	1 (0.5)	0 (0.0)	−0.059
Diabetes mellitus	159 (23.9)	131 (23.0)	−0.021	519 (20.0)	272 (20.5)	0.012	186 (26.1)	270 (29.0)	0.067	31 (15.4)	79 (25.3)	0.248
Previous stroke/TIA/systemic embolism	103 (15.5)	63 (11.1)	−0.130	423 (16.3)	196 (14.7)	−0.043	93 (13.0)	104 (11.2)	−0.057	26 (12.9)	38 (12.2)	−0.023
Myocardial infarction	38 (5.7)	17 (3.0)	−0.134	230 (8.9)	117 (8.8)	−0.002	71 (9.9)	84 (9.0)	−0.031	10 (5.0)	21 (6.7)	0.075
Unknown	0 (0.0)	0 (0.0)	0.000	2 (0.1)	0 (0.0)	−0.017	1 (0.1)	2 (0.2)	0.018	0 (0.0)	1 (0.3)	0.014
Coronary artery disease	117 (17.6)	50 (8.8)	−0.262	419 (16.1)	245 (18.4)	0.061	199 (27.9)	224 (24.1)	−0.086	20 (10.0)	32 (10.3)	0.010
Unknown	15 (2.3)	0 (0.0)	−0.202	78 (3.0)	48 (3.6)	0.034	13 (1.8)	6 (0.6)	−0.107	6 (3.0)	3 (1.0)	−0.146
Vascular disease	50 (7.5)	22 (3.9)	−0.158	313 (12.0)	156 (11.7)	−0.010	94 (13.2)	122 (13.1)	−0.001	15 (7.5)	27 (8.7)	0.044
Cancer	62 (9.3)	23 (4.0)	−0.213	204 (7.9)	131 (9.8)	0.070	124 (17.4)	135 (14.5)	−0.078	6 (3.0)	11 (3.5)	0.031
Unknown	10 (1.5)	1 (0.2)	−0.146	33 (1.3)	25 (1.9)	0.049	2 (0.3)	1 (0.1)	−0.039	5 (2.5)	1 (0.3)	−0.185
Chronic gastrointestinal disease	146 (21.9)	46 (8.1)	−0.396	220 (8.5)	95 (7.1)	−0.049	145 (20.3)	197 (21.2)	0.022	31 (15.4)	24 (7.7)	−0.244
Unknown	16 (2.4)	0 (0.0)	−0.210	41 (1.6)	27 (2.0)	0.034	3 (0.4)	0 (0.0)	−0.075	1 (0.5)	0 (0.0)	−0.059
Hepatic disease	43 (6.5)	14 (2.5)	−0.195	29 (1.1)	9 (0.7)	−0.047	10 (1.4)	8 (0.9)	–0.051	4 (2.0)	1 (0.3)	−0.157
Unknown	11 (1.7)	1 (0.2)	−0.156	47 (1.8)	32 (2.4)	0.042	5 (0.7)	1 (0.1)	−0.094	9 (4.5)	0 (0.0)	−0.281
Chronic kidney disease^b^	216 (32.4)	155 (27.2)	−0.115	623 (24.0)	302 (22.7)	−0.030	135 (18.9)	171 (18.4)	−0.013	39 (19.4)	64 (20.5)	0.028
Unknown	119 (17.9)	141 (24.7)	0.168	505 (19.4)	173 (13.0)	−0.175	186 (26.1)	177 (19.0)	−0.167	91 (45.3)	145 (46.5)	0.024
Prior bleeding	40 (6.0)	18 (3.2)	−0.137	118 (4.5)	53 (4.0)	−0.028	42 (5.9)	42 (4.5)	−0.062	8 (4.0)	11 (3.5)	−0.024
Unknown	14 (2.1)	0 (0.0)	−0.195	61 (2.3)	48 (3.6)	0.074	16 (2.2)	10 (1.1)	−0.091	2 (1.0)	0 (0.0)	−0.110
Type of site, n (%)												
GP/primary care	48 (7.2)	0 (0.0)	−0.387	78 (3.0)	16 (1.2)	−0.126	68 (9.5)	63 (6.8)	−0.100	39 (19.4)	66 (21.2)	0.044
Specialist office	66 (9.9)	37 (6.5)	−0.125	526 (20.2)	348 (26.2)	0.141	557 (78.0)	677 (72.8)	−0.121	70 (34.8)	102 (32.7)	−0.046
Community hospital	385 (57.8)	49 (8.6)	−1.226	1120 (43.1)	652 (49.0)	0.119	48 (6.7)	91 (9.8)	0.112	71 (35.3)	65 (20.8)	−0.327
University hospital	154 (23.1)	484 (84.9)	1.580	785 (30.2)	280 (21.1)	−0.211	32 (4.5)	97 (10.4)	0.228	16 (8.0)	49 (15.7)	0.242
Other^c^	13 (2.0)	0 (0.0)	−0.186	89 (3.4)	34 (2.6)	−0.051	9 (1.3)	2 (0.2)	−0.122	5 (2.5)	30 (9.6)	0.302
Physician specialty, n (%)												
GP/PCP/geriatrician	13 (2.0)	1 (0.2)	−0.174	50 (1.9)	22 (1.7)	−0.020	18 (2.5)	33 (3.5)	0.060	13 (6.5)	18 (5.8)	−0.029
Cardiologist	649 (97.4)	554 (97.2)	−0.016	2279 (87.7)	1183 (88.9)	0.038	622 (87.1)	801 (86.1)	−0.029	187 (93.0)	292 (93.6)	0.022
Neurologist	0 (0.0)	14 (2.5)	0.214	118 (4.5)	95 (7.1)	0.111	22 (3.1)	17 (1.8)	−0.081	0 (0.0)	0 (0.0)	0.000
Internist	4 (0.6)	1 (0.2)	−0.068	61 (2.3)	16 (1.2)	−0.087	49 (6.9)	58 (6.2)	−0.025	1 (0.5)	2 (0.6)	0.019
Angiologist	0 (0.0)	0 (0.0)	0.000	0 (0.0)	0 (0.0)	0.000	0 (0.0)	0 (0.0)	0.000	0 (0.0)	0 (0.0)	0.000
Other	0 (0.0)	0 (0.0)	0.000	90 (3.5)	14 (1.1)	−0.163	3 (0.4)	21 (2.3)	0.160	0 (0.0)	0 (0.0)	0.000
Medical treatment reimbursed by, n (%)												
Self‐pay/no coverage	66 (9.9)	106 (18.6)	0.250	105 (4.0)	21 (1.6)	−0.149	13 (1.8)	17 (1.8)	0.001	56 (27.9)	79 (25.3)	−0.058
Not self‐pay^d^	581 (87.2)	415 (72.8)	−0.367	2329 (89.6)	1263 (95.0)	0.201	669 (93.7)	881 (94.7)	0.044	137 (68.2)	225 (72.1)	0.087
Unknown	19 (2.9)	49 (8.6)	0.249	164 (6.3)	46 (3.5)	−0.133	32 (4.5)	32 (3.4)	−0.053	8 (4.0)	8 (2.6)	−0.080

Abbreviations: AF, atrial fibrillation; BMI, body mass index; CHA_2_DS_2_‐VASc, congestive heart failure, hypertension, age ≥75 years, diabetes, stroke/transient ischemic attack/systemic embolism, vascular disease, age 65‐74 years, sex category (female); GP, general practitioner; HAS‐BLED, hypertension, abnormal renal/liver function, stroke, bleeding history or predisposition, labile international normalized ratio, elderly (>65 years), drugs or alcohol concomitantly; IQR, interquartile range; NOAC, non‐Vitamin K antagonist oral anticoagulant; PCP, primary care physician; TIA, transient ischemic attack.

^a^ ≥ 8 units/wk.

^b^ < 60 mL/min.

^c^ Anticoagulation clinics, out‐patient healthcare centers, and other healthcare settings.

^d^ Private and statutory/ federal insurance.

Baseline characteristics of patients prescribed VKA by region are shown in Table 2. The standardized differences for CHA_2_DS_2_‐VASc were small between the patients enrolled during the last and first year in North America, while they were more than 0.1 in Europe, Asia and Latin America. The standardized differences for HAS‐BLED were more than 0.1 between last and first year in Asia, North America, and Latin America.

**TABLE 2:** Baseline characteristics for VKA patients by first and last year of enrollment by region

Characteristic	Asia			Europe			North America			Latin America		
	VKA during first year (n = 594)	VKA during last year (n = 92)	Std diff	VKA during first year (n = 1728)	VKA during last year (n = 295)	Std diff	VKA during first year (n = 422)	VKA during last year (n = 152)	Std diff	VKA during first year (n = 117)	VKA during last year (n = 78)	Std diff
Age, median, IQR, y	68.0 (60.0‐75.0)	63.5 (56.0‐71.5)	–0.418	74.0 (66.0‐79.5)	75.0 (68.0‐80.0)	0.127	73.0 (65.0‐81.0)	72.5 (63.5‐79.0)	–0.100	70.0 (62.0‐77.0)	69.0 (63.0‐76.0)	0.021
Females, n (%)	259 (43.6)	32 (34.8)	–0.181	819 (47.4)	157 (53.2)	0.117	202 (47.9)	68 (44.7)	–0.063	46 (39.3)	30 (38.5)	−0.018
Alcohol abuse, n (%)^a^	46 (7.7)	4 (4.3)	–0.143	156 (9.0)	8 (2.7)	–0.271	21 (5.0)	6 (3.9)	–0.050	0 (0.0)	6 (7.7)	0.375
Unknown	68 (11.4)	5 (5.4)	–0.218	112 (6.5)	22 (7.5)	0.038	18 (4.3)	6 (3.9)	–0.016	3 (2.6)	2 (2.6)	0.000
BMI, median, IQR, kg/m^2^	24.7 (22.7‐27.1)	24.8 (22.7‐27.6)	0.097	28.0 (25.0‐31.6)	28.0 (25.0‐32.4)	0.054	30.0 (26.4‐35.5)	31.2 (26.5‐35.8)	0.004	28.1 (25.6‐31.6)	27.2 (24.4‐31.9)	0.092
Missed, n (%)	22 (3.7)	1 (1.1)		20 (1.2)	10 (3.4)		0 (0.0)	0 (0.0)		1 (0.9)	0 (0.0)	
Type of AF, n (%)												
Paroxysmal	294 (49.5)	60 (65.2)	0.322	689 (39.9)	126 (42.7)	0.058	245 (58.1)	107 (70.4)	0.260	42 (35.9)	32 (41.0)	0.106
Persistent	275 (46.3)	23 (25.0)	–0.456	792 (45.8)	135 (45.8)	–0.001	150 (35.5)	41 (27.0)	–0.186	44 (37.6)	21 (26.9)	−0.230
Permanent	25 (4.2)	9 (9.8)	0.220	247 (14.3)	34 (11.5)	–0.083	27 (6.4)	4 (2.6)	–0.182	31 (26.5)	25 (32.1)	0.122
Categorization of AF, n (%)												
Symptomatic	135 (22.7)	19 (20.7)	–0.050	532 (30.8)	122 (41.4)	0.221	97 (23.0)	45 (29.6)	0.151	31 (26.5)	31 (39.7)	0.284
Minimally symptomatic	297 (50.0)	43 (46.7)	–0.065	661 (38.3)	76 (25.8)	–0.270	162 (38.4)	50 (32.9)	–0.115	44 (37.6)	18 (23.1)	−0.320
Asymptomatic	162 (27.3)	30 (32.6)	0.117	535 (31.0)	97 (32.9)	0.041	163 (38.6)	57 (37.5)	–0.023	42 (35.9)	29 (37.2)	0.027
Creatinine clearance (measured), median, IQR, ml/min	69.1 (52.1‐86.3)	76.0 (57.5‐96.9)	0.382	72.0 (52.8‐94.5)	68.9 (47.5‐89.3)	–0.098	73.7 (50.4‐109.7)	80.5 (52.4‐110.6)	0.077	74.1 (57.9‐95.7)	67.0 (52.6‐93.7)	–0.023
<15, n (%)	9 (1.5)	0 (0.0)	–0.097	18 (1.0)	2 (0.7)	–0.039	5 (1.2)	1 (0.7)	–0.055	0 (0.0)	0 (0,0)	0.000
15‐29, n (%)	20 (3.4)	0 (0.0)	–0.205	52 (3.0)	25 (8.5)	0.237	14 (3.3)	8 (5.3)	0.096	3 (2.6)	2 (2.6)	0.000
30‐49, n (%)	73 (12.3)	10 (10.9)	–0.044	231 (13.4)	40 (13.6)	0.006	61 (14.5)	20 (13.2)	–0.038	10 (8.5)	4 (5.1)	−0.136
50‐79, n (%)	215 (36.2)	32 (34.8)	–0.030	533 (30.8)	84 (28.5)	–0.052	96 (22.7)	33 (21.7)	–0.025	23 (19.7)	23 (29.5)	0.223
≥80, n (%)	148 (24.9)	31 (33.7)	0.194	566 (32.8)	86 (29.2)	–0.078	148 (35.1)	67 (44.1)	0.185	28 (23.9)	17 (21.8)	−0.051
Missing, n (%)	129 (21.7)	19 (20.7)	–0.026	328 (19.0)	58 (19.7)	0.017	98 (23.2)	23 (15.1)	–0.207	53 (45.3)	32 (41.0)	−0.086
CHA_2_DS_2_‐VASc score, median, IQR	3.0 (2.0‐4.0)	2.0 (1.0‐3.0)	–0.521	3.0 (2.0‐4.0)	4.0 (3.0‐4.0)	0.149	4.0 (2.0‐4.0)	3.0 (3.0‐4.0)	–0.025	3.0 (2.0‐4.0)	3.0 (2.0‐4.0)	–0.181
HAS‐BLED score, median, IQR	1.0 (1.0‐2.0)	1.0 (0.0‐1.0)	–0.389	1.0 (1.0‐2.0)	1.0 (1.0‐2.0)	–0.069	1.0 (1.0‐2.0)	1.0 (1.0‐2.0)	0.123	1.0 (0.0‐2.0)	1.0 (0.0‐1.0)	–0.289
Missing (HAS‐BLED), n (%)	95 (16.0)	6 (6.5)	−0.303	204 (11.8)	38 (12.9)	0.033	50 (11.8)	10 (6.6)	−0.183	20 (17.1)	2 (2.6)	−0.503
Medical history, n (%)												
Congestive heart failure	117 (19.7)	30 (32.6)	0.297	435 (25.2)	76 (25.8)	0.014	111 (26.3)	55 (36.2)	0.214	50 (42.7)	26 (33.3)	−0.195
Unknown	5 (0.8)	0 (0.0)	–0.036	30 (1.7)	4 (1.4)	–0.031	4 (0.9)	2 (1.3)	0.035	1 (0.9)	0 (0.0)	−0.025
Hypertension	402 (67.7)	52 (56.5)	–0.232	1273 (73.7)	225 (76.3)	0.060	352 (83.4)	134 (88.2)	0.136	89 (76.1)	59 (75.6)	−0.010
Unknown	1 (0.2)	0 (0.0)	0.063	8 (0.5)	0 (0.0)	–0.052	0 (0.0)	0 (0.0)	0.000	0 (0.0)	0 (0.0)	0.000
Diabetes mellitus	135 (22.7)	17 (18.5)	–0.105	385 (22.3)	97 (32.9)	0.239	136 (32.2)	57 (37.5)	0.111	20 (17.1)	16 (20.5)	0.088
Unknown	0 (0.0)	0 (0.0)	0.000	0 (0.0)	0 (0.0)	0.000	0 (0.0)	0 (0.0)	0.000	0 (0.0)	0 (0.0)	0.000
Previous stroke/TIA/systemic embolism	91 (15.3)	8 (8.7)	–0.205	267 (15.5)	33 (11.2)	–0.126	64 (15.2)	20 (13.2)	−0.058	16 (13.7)	5 (6.4)	−0.243
Unknown	0 (0.0)	0 (0.0)	0.000	0 (0.0)	0 (0.0)	0.000	0 (0.0)	0 (0.0)	0.000	101 (86.3)	73 (93.6)	0.244
Myocardial infarction	15 (2.5)	2 (2.2)	–0.023	246 (14.2)	38 (12.9)	–0.040	58 (13.7)	19 (12.5)	−0.037	15 (12.8)	5 (6.4)	−0.219
Unknown	0 (0.0)	0 (0.0)	0.000	1 (0.1)	0 (0.0)	0.033	0 (0.0)	0 (0.0)	0.000	0 (0.0)	0 (0.0)	0.000
Coronary artery disease	70 (11.8)	14 (15.2)	0.101	323 (18.7)	49 (16.6)	–0.055	131 (31.0)	48 (31.6)	0.012	12 (10.3)	7 (9.0)	−0.044
Unknown	6 (1.0)	0 (0.0)	–0.054	82 (4.7)	23 (7.8)	0.126	6 (1.4)	1 (0.7)	−0.075	4 (3.4)	0 (0.0)	−0.198
Vascular disease	22 (3.7)	2 (2.2)	–0.091	311 (18.0)	52 (17.6)	–0.010	77 (18.2)	27 (17.8)	−0.013	17 (14.5)	9 (11.5)	−0.089
Cancer	33 (5.6)	2 (2.2)	–0.176	135 (7.8)	25 (8.5)	0.024	66 (15.6)	33 (21.7)	0.156	4 (3.4)	2 (2.6)	−0.050
Unknown	5 (0.8)	0 (0.0)	–0.036	8 (0.5)	10 (3.4)	0.214	2 (0.5)	0 (0.0)	−0.023	1 (0.9)	0 (0.0)	−0.025
Chronic gastrointestinal disease	64 (10.8)	6 (6.5)	–0.152	168 (9.7)	36 (12.2)	0.080	92 (21.8)	36 (23.7)	0.045	7 (6.0)	4 (5.1)	−0.037
Unknown	5 (0.8)	0 (0.0)	–0.036	17 (1.0)	9 (3.1)	0.147	2 (0.5)	0 (0.0)	−0.023	2 (1.7)	1 (1.3)	−0.035
Hepatic disease	14 (2.4)	2 (2.2)	–0.012	27 (1.6)	1 (0.3)	–0.126	9 (2.1)	4 (2.6)	0.033	0 (0.0)	0 (0.0)	0.000
Unknown	7 (1.2)	1 (1.1)	–0.009	19 (1.1)	3 (1.0)	–0.008	6 (1.4)	0 (0.0)	−0.1177	12 (10.3)	0 (0.0)	−0.434
Chronic kidney disease^b^	166 (27.9)	22 (23.9)	–0.092	484 (28.0)	85 (28.8)	0.018	122 (28.9)	44 (28.9)	0.001	18 (15.4)	19 (24.4)	0.226
Unknown	129 (21.7)	19 (20.7)	–0.026	328 (19.0)	58 (19.7)	0.017	98 (23.2)	23 (15.1)	−0.207	53 (45.3)	32 (41.0)	−0.086
Prior bleeding	28 (4.7)	3 (3.3)	–0.074	90 (5.2)	12 (4.1)	–0.054	34 (8.1)	17 (11.2)	0.106	7 (6.0)	2 (2.6)	−0.170
Unknown	10 (1.7)	0 (0.0)	–0.109	23 (1.3)	10 (3.4)	0.136	16 (3.8)	1 (0.7)	−0.214	0 (0.0)	0 (0.0)	0.000
Type of site, n (%)												
GP/primary care	5 (0.8)	0 (0.0)	–0.036	62 (3.6)	1 (0.3)	–0.236	55 (13.0)	12 (7.9)	−0.169	9 (7.7)	8 (10.3)	0.090
Specialist office	83 (14.0)	15 (16.3)	0.065	193 (11.2)	75 (25.4)	0.375	295 (69.9)	93 (61.2)	−0.184	35 (29.9)	30 (38.5)	0.181
Community hospital	43 (7.2)	9 (9.8)	0.091	695 (40.2)	120 (40.7)	0.009	35 (8.3)	14 (9.2)	0.032	35 (29.9)	6 (7.7)	−0.593
University hospital	460 (77.4)	69 (73.9)	–0.082	714 (41.3)	99 (33.6)	–0.161	27 (6.4)	28 (18.4)	0.371	34 (29.1)	33 (42.3)	0.279
Other^c^	3 (0.5)	0 (0.0)	0.005	64 (3.7)	0 (0.0)	–0.259	10 (2.4)	5 (3.3)	0.056	4 (3.4)	1 (1.3)	−0.141
Physician specialty, n (%)												
GP/PCP/geriatrician	1 (0.2)	0 (0.0)	0.063	68 (3.9)	2 (0.7)	–0.218	21 (5.0)	10 (6.6)	0.069	1 (0.9)	0 (0.0)	−0.025
Cardiologist	592 (99.7)	92 (100.0)	–0.031	1385 (80.2)	281 (95.3)	0.473	360 (85.3)	122 (80.3)	−0.134	116 (99.1)	77 (98.7)	−0.042
Neurologist	1 (0.2)	0 (0.0)	0.063	32 (1.9)	9 (3.1)	0.078	5 (1.2)	6 (3.9)	0.175	0 (0.0)	0 (0.0)	0.000
Internist	0 (0.0)	0 (0.0)	0.000	57 (3.3)	3 (1.0)	–0.158	35 (8.3)	12 (7.9)	−0.015	0 (0.0)	1 (1.3)	0.093
Angiologist	0 (0.0)	0 (0.0)	0.000	0 (0.0)	0 (0.0)	0.000	0 (0.0)	0 (0.0)	0.000	0 (0.0)	0 (0.0)	0.000
Other	0 (0.0)	0 (0.0)	0.000	186 (10.8)	0 (0.0)	–0.479	1 (0.2)	2 (1.3)	0.123	0 (0.0)	0 (0.0)	0.000
Medical treatment reimbursed by, n (%)												
Self‐pay/no coverage	69 (11.6)	5 (5.4)	–0.223	108 (6.3)	7 (2.4)	–0.192	14 (3.3)	8 (5.3)	0.096	13 (11.1)	10 (12.8)	0.053
Not self‐pay^d^	510 (85.9)	76 (82.6)	–0.089	1437 (83.2)	284 (96.3)	0.442	376 (89.1)	136 (89.5)	0.012	94 (80.3)	60 (76.9)	−0.084
Unknown	15 (2.5)	11 (12.0)	0.370	183 (10.6)	4 (1.4)	–0.397	32 (7.6)	8 (5.3)	−0.095	10 (8.5)	8 (10.3)	0.059

Abbreviations: AF, atrial fibrillation; BMI, body mass index; CHA_2_DS_2_‐VASc, congestive heart failure, hypertension, age ≥75 years, diabetes, stroke/transient ischemic attack/systemic embolism, vascular disease, age 65‐74 years, sex category (female); GP, general practitioner; HAS‐BLED, hypertension, abnormal renal/liver function, stroke, bleeding history or predisposition, labile international normalized ratio, elderly (>65 years), drugs or alcohol concomitantly; IQR, interquartile range; PCP, primary care physician; TIA, transient ischemic attack; VKA, Vitamin K antagonists.

^a^ ≥ 8 units/wk.

^b^ < 60 mL/min.

^c^ Anticoagulation clinics, out‐patient healthcare centers, and other healthcare settings.

^d^ Private and statutory/ federal insurance.

Prescription of oral antithrombotic treatment by region is presented in Table 3 and Figure 3. A decrease in no OAC use including acetylsalicylic acid (ASA) was reported in Asia between the third and fourth year of enrolment. An increase in NOAC use and decrease in VKA and no OAC use including ASA was present in Europe and North America. An increase in NOAC and a decrease in VKA were reported between second and fourth year in Latin America.

**TABLE 3:** Prescription of oral antithrombotic treatment over time by region

	Year 1	Year 2	Year 3	Year 4	Total
Region: Asia					
Number of patients	2284 (100.0)	1336 (100.0)	1396 (100.0)	937 (100.0)	5953 (100.0)
NOAC (N, %)	666 (29.2)	619 (46.3)	527 (37.8)	570 (60.8)	2382 (40.0)
On NOACs standard dose (N, %)					
Yes	214 (9.4)	239 (17.9)	204 (14.6)	224 (23.9)	881 (14.8)
No	452 (19.8)	380 (28.4)	323 (23.1)	346 (36.9)	1501 (25.2)
On NOACs reduced dose (N, %)					
Yes	452 (19.8)	380 (28.4)	323 (23.1)	346 (36.9)	1501 (25.2)
No	214 (9.4)	239 (17.9)	204 (14.6)	224 (23.9)	881 (14.8)
VKA (N, %)	594 (26.0)	266 (19.9)	330 (23.6)	92 (9.8)	1282 (21.5)
No OAC (N, %)	1024 (44.8)	451 (33.8)	539 (38.6)	275 (29.3)	2289 (38.5)
ASA (N, %)	522 (22.9)	251 (18.8)	313 (22.4)	150 (16.0)	1236 (20.8)
Antiplts other than ASA (N, %)	34 (1.5)	22 (1.6)	35 (2.5)	16 (1.7)	107 (1.8)
None (N, %)	468 (20.5)	178 (13.3)	191 (13.7)	109 (11.6)	946 (15.9)
Region: Europe					
Number of patients	4866 (100.0)	4090 (100.0)	2911 (100.0)	1754 (100.0)	13 621 (100.0)
NOAC (N, %)	2598 (53.4)	2308 (56.4)	1899 (65.2)	1330 (75.8)	8135 (59.7)
On NOACs standard dose (N, %)					
Yes	1523 (31.3)	1514 (37.0)	1373 (47.2)	1016 (57.9)	5426 (39.8)
No	1075 (22.1)	794 (19.4)	526 (18.1)	314 (17.9)	2709 (19.9)
On NOACs reduced dose (N, %)					
Yes	1075 (22.1)	794 (19.4)	526 (18.1)	314 (17.9)	2709 (19.9)
No	1523 (31.3)	1514 (37.0)	1373 (47.2)	1016 (57.9)	5426 (39.8)
VKA (N, %)	1728 (35.5)	1367 (33.4)	741 (25.5)	295 (16.8)	4131 (30.3)
No OAC (N, %)	540 (11.1)	415 (10.1)	271 (9.3)	129 (7.4)	1355 (9.9)
ASA (N, %)	280 (5.8)	228 (5.6)	115 (4.0)	66 (3.8)	689 (5.1)
Antiplts other than ASA (N, %)	43 (0.9)	36 (0.9)	22 (0.8)	4 (0.2)	105 (0.8)
None (N, %)	217 (4.5)	151 (3.7)	134 (4.6)	59 (3.4)	561 (4.1)
Region: North America					
Number of patients	1458 (100.0)	2045 (100.0)	1593 (100.0)	1258 (100.0)	6354 (100.0)
NOAC (N, %)	714 (49.0)	1215 (59.4)	1093 (68.6)	930 (73.9)	3952 (62.2)
On NOACs standard dose (N, %)					
Yes	599 (41.1)	1033 (50.5)	943 (59.2)	772 (61.4)	3347 (52.7)
No	115 (7.9)	182 (8.9)	150 (9.4)	158 (12.6)	605 (9.5)
On NOACs reduced dose (N, %)					
Yes	115 (7.9)	182 (8.9)	150 (9.4)	158 (12.6)	605 (9.5)
No	599 (41.1)	1033 (50.5)	943 (59.2)	772 (61.4)	3347 (52.7)
VKA (N, %)	422 (28.9)	442 (21.6)	216 (13.6)	152 (12.1)	1232 (19.4)
No OAC (N, %)	322 (22.1)	388 (19.0)	284 (17.8)	176 (14.0)	1170 (18.4)
ASA (N, %)	200 (13.7)	262 (12.8)	200 (12.6)	134 (10.7)	796 (12.5)
Antiplts other than ASA (N, %)	4 (0.3)	21 (1.0)	5 (0.3)	4 (0.3)	34 (0.5)
None (N, %)	118 (8.1)	105 (5.1)	79 (5.0)	38 (3.0)	340 (5.4)
Region: Latin America					
Number of patients	361 (100.0)	420 (100.0)	284 (100.0)	439 (100.0)	1504 (100.0)
NOAC (N, %)	201 (55.7)	219 (52.1)	164 (57.7)	312 (71.1)	896 (59.6)
On NOACs standard dose (N, %)					
Yes	122 (33.8)	112 (26.7)	83 (29.2)	142 (32.3)	459 (30.5)
No	79 (21.9)	107 (25.5)	81 (28.5)	170 (38.7)	437 (29.1)
On NOACs reduced dose (N, %)					
Yes	79 (21.9)	107 (25.5)	81 (28.5)	170 (38.7)	437 (29.1)
No	122 (33.8)	112 (26.7)	83 (29.2)	142 (32.3)	459 (30.5)
VKA (N, %)	117 (32.4)	137 (32.6)	73 (25.7)	78 (17.8)	405 (26.9)
No OAC (N, %)	43 (11.9)	64 (15.2)	47 (16.5)	49 (11.2)	203 (13.5)
ASA (N, %)	24 (6.6)	46 (11.0)	33 (11.6)	31 (7.1)	134 (8.9)
Antiplts other than ASA (N, %)	3 (0.8)	4 (1.0)	3 (1.1)	3 (0.7)	13 (0.9)
None (N, %)	16 (4.4)	14 (3.3)	11 (3.9)	15 (3.4)	56 (3.7)

Note

Standard dose: Dabigatran 150‐mg BID, Rivaroxaban 20‐mg QD, Apixaban 5‐mg BID, Edoxaban 60‐mg QD. The other doses are reduced.

Abbreviations: ASA, acetylsalycylic acid, NOAC, non‐vitamin K antagonist oral anticoagulants, VKA, Vitamin K antagonists.

### 3.1 Factors associated with NOAC prescription in phases II and III

Results from univariate analyses are presented in Table 4. In the multivariable log‐binomial regression analysis, factors strongly associated with *increased* prescription of NOAC were as follows: enrolment year, type of site (higher probability outside of a university hospital, such as GP/primary care, specialist office, community hospital, and other), and region (higher prescription probability in North America compared with Europe) (Table 4).

**TABLE 4:** Multivariable log‐binomial analysis for factors associated with prescription of oral antithrombotic therapy (NOAC versus VKA)

Variable	Total N (100%)	NOAC n (%)	VKA n (%)	Univariate analysis relative proportion (95% CI) for NOAC prescription	Multivariate analysis relative proportion (95% CI) for NOAC prescription
Time (categorical, Years 1‐4)					
Year 1	7040	4179 (59.4)	2861 (40.6)	1.0 (ref)	1.0 (ref)
Year 2	6573	4361 (66.3)	2212 (33.7)	1.118 (1.09‐1.15)	1.10 (1.07‐1.12)
Year 3	5043	3683 (73.0)	1360 (27.0)	1.23 (1.20‐1.26)	1.20 (1.17‐1.23)
Year 4	3759	3142 (83.6)	617 (16.4)	1.41 (1.38‐1.44)	1.34 (1.31‐1.37)
Region					
Asia	3664	2382 (65.0)	1282 (35.0)	0.98 (0.95‐1.01)	1.03 (0.99‐1.05)
Europe	12 266	8135 (66.3)	4131 (33.7)	1.0 (ref)	1.0 (ref)
North America	5184	3952 (76.2)	1232 (23.8)	1.15 (1.13‐1.17)	1.05 (1.03‐1.08)
Latin America	1301	896 (68.9)	405 (31.1)	1.04 (0.99‐1.08)	0.99 (0.96‐1.04)
BMI class					
<18.5	297	201 (67.7)	96 (32.3)	0.99 (0.92‐1.09)	0.98 (0.91‐1.05)
18.5‐24	5856	3970 (67.8)	1887 (32.2)	1.0 (ref)	1.0 (ref)
25‐29	8623	5884 (68.2)	2738 (31.8)	1.01 (0.98‐1.03)	1.00 (0.98‐1.02)
30‐34	4582	3165 (69.1)	1417 (30.9)	1.02 (0.99‐1.05)	0.99 (0.97‐1.01)
≥35	3057	2145 (70.2)	912 (29.8)	1.04 (1.01‐1.07)	0.98 (0.96‐1.01)
Categorization of AF					
Symptomatic	6996	4740 (67.8)	2256 (32.2)	0.96 (0.94‐0.98)	0.98 (0.96‐0.99)
Minimally symptomatic	7915	5332 (67.4)	2583 (32.6)	0.96 (0.94‐0.98)	0.99 (0.97‐1.00)
Asymptomatic	7504	5293 (70.5)	2211 (29.5)	1.0 (ref)	1.0 (ref)
HAS‐BLED score <3	20 619	14 176 (68.8)	6443 (31.2)	1.0 (ref)	1.0 (ref)
HAS‐BLED score ≥3	1796	1189 (66.2)	607 (33.8)	0.96 (0.93‐0.99)	0.96 (0.93‐0.99)
CHA_2_DS_2_‐VASc score =1	2741	1902 (69.4)	839 (30.6)	1.0 (ref)	1.0 (ref)
CHA_2_DS_2_‐VASc score ≥2	19 674	13 463 (68.4)	6211 (31.6)	0.99 (0.96‐1.01)	0.97 (0.95‐0.99)
Chronic gastrointestinal disease					
Yes	2968	2101 (70.8)	867 (29.2)	1.04 (1.01‐1.06)	1.01 (0.99‐1.03)
No	19 447	13 264 (68.2)	6183 (31.8)	1.0 (ref)	1.0 (ref)
Type of AF					
Paroxysmal	11 828	8536 (72.2)	3292 (27.8)	1.0 (ref)	1.0 (ref)
Persistent	8239	5304 (64.4)	2935 (35.6)	0.89 (0.88‐0.91)	0.93 (0.92‐0.95)
Permanent	2348	1525 (64.9)	823 (35.1)	0.90 (0.87‐0.93)	0.93 (0.90‐0.96)
Type of site					
GP/primary care	1123	830 (73.9)	293 (26.1)	1.282 (1.230‐1.334)	1.24 (1.19‐1.29)
Specialist office	6945	5199 (74.9)	1746 (25.1)	1.29 (1.27‐1.33)	1.22 (1.19‐1.25)
Community hospital	7205	5171 (71.8)	2034 (28.2)	1.25 (1.21‐1.28)	1.23 (1.20‐1.27)
University hospital	6540	3769 (57.6)	2771 (42.4)	1.0 (ref)	1.0 (ref)
Other	602	396 (65.8)	206 (34.2)	1.14 (1.07‐1.21)	1.15 (1.09‐1.22)
Cancer					
Yes	2261	1586 (70.1)	675 (29.9)	1.026 (0.99‐1.06)	0.99 (0.97‐1.02)
No	20 154	13 779 (68.4)	6375 (31.6)	1.0 (ref)	1.0 (ref)
Medical treatment reimbursed by					
Self‐pay/no overage	1362	894 (65.6)	467 (34.3)	0.956 (0.92‐0.99)	0.99 (0.96‐1.03)
Not self‐pay	21 053	14 471 (68.7)	6583 (31.3)	1.0 (ref)	1.0 (ref)

Abbreviations: AF, atrial fibrillation; BMI, body mass index; CHA_2_DS_2_‐VASc, congestive heart failure, hypertension, age ≥75 years, diabetes, stroke/transient ischemic attack/systemic embolism, vascular disease, age 65‐74 years, sex category (female); CI, confidence interval; GP, general practitioner; HAS‐BLED, hypertension, abnormal renal/liver function, stroke, bleeding history or predisposition, labile international normalized ratio, elderly (>65 years), drugs or alcohol concomitantly; NOAC, nonvitamin K antagonist oral anticoagulants; ref, reference; VKA, Vitamin K antagonist.

Factors associated with *decreased* prescription of NOAC were the following: HAS‐BLED score ≥3 (compared with HAS‐BLED score <3), categorization of AF (lower probability of symptomatic AF compared with asymptomatic AF), CHA_2_DS_2_‐VASc score ≥2 (compared with CHA_2_DS_2_‐VASc score <2), and type of AF (lower probability of persistent or permanent AF compared with paroxysmal AF) (Table 4).

### 3.2 Prescription of antithrombotics over time by CHA_2_DS_2_‐VASc score class

Regional patterns of prescription of antithrombotics over time by CHA_2_DS_2_‐VASc score class are presented in Table S1.

In the first year after approval, 32.5% of those with CHA_2_DS_2_‐VASc scores ≥2 received NOACs in Asia. Corresponding proportions for patients in Europe, North America, and Latin America were 53.5%, 49.6%, and 56.2%. In the fourth year after approval, 67.1% of patients with CHA_2_DS_2_‐VASc scores ≥2 received NOACs in Asia. Corresponding proportions for patients in Europe, North America, and Latin America were 75.7%, 75.4%, and 70.4%.

Let *interval change* denote the change between Year 4 and Year 1 in prescription rate. The interval changes in those with CHA_2_DS_2_‐VASc scores ≥2 who received NOACs in Asia were +34.6%. The corresponding interval changes for Europe, North America, and Latin America were +22.2%, +25.8%, and +14.2%. The interval changes in those with CHA_2_DS_2_‐VASc scores ≥2 who received VKAs in Asia were −17.5%. The corresponding interval changes for Europe, North America, and Latin America were −18.5%, −17.2%, and −15.3% (Table S2).

### 3.3 Prescription of antithrombotics over time by HAS‐BLED score class

Regional patterns of prescription of antithrombotic drugs over time by HAS‐BLED score class are presented in Table S3. The interval changes in those with HAS‐BLED scores ≥3 who received NOACs in Asia were +4.1%. The corresponding interval changes for Europe, North America and Latin America were +20.7, +20.3, and +22.5%. The interval changes in those with HAS‐BLED scores ≥3 who received VKAs in Asia were −9.7%. The corresponding interval changes for Europe, North America, and Latin America were −18.5%, −17.2%, and −21.3% (Table S4).

## 4 DISCUSSION

We found that use of NOAC increased and VKA decreased over time in patients with newly diagnosed AF. In consecutive years after their introduction, the proportions of patients prescribed NOAC increased and exceeded that of VKA or no OAC in all geographical regions, just as prescriptions for VKA decreased in all regions. *North America was associated with NOAC prescription in the univariate analysis of NOAC vs. VKA prescription*. The interval changes between fourth and first year after NOAC approval regarding NOAC prescription in patients with CHA_2_DS_2_‐VASc scores ≥2 were the highest in Asia and North America. The interval changes between fourth and first year after NOAC approval regarding NOAC prescription in patients with HAS‐BLED score ≥3 were the highest in Latin America, Europe, and North America. The prescription of non‐OAC agents decreased in Asia, Europe, and North America but remained little changed in Latin America.

The use of NOAC appears to have increased over time in Europe. This finding is consistent with other reports.^7^, ^8^, ^9^ After the release of NOAC, the prevalence of NOAC use rose steadily in Japan.^10^ Similar patterns of NOAC and VKA prescription were shown in Outcomes Registry for Better Informed Treatment of Atrial Fibrillation (ORBIT‐AF).^11^

Smaller proportions of patients from Latin America were prescribed NOAC or VKA at baseline in the Global Anticoagulant Registry in the Field‐Atrial Fibrillation (GARFIELD‐AF) than in our registry.^12^ Patients prescribed VKA during the last year of enrolment were more likely to have concomitant diseases, such as CHF, diabetes or vascular disease, than those who use NOAC during their last year of enrolment in Europe. In other studies, patients prescribed VKAs also had more comorbidities than those prescribed NOACs.^13^, ^14^ Similar to our study, patients prescribed VKA were more likely to have permanent AF than those prescribed NOAC in each region.^13^, ^14^ Interestingly, in Korean patients those who used VKAs were less likely to have prior stroke/TIA/systemic embolism than those who used NOACs.^15^

In our study, the proportion of patients who were prescribed a reduced dose of NOAC is highest in Asia, a finding that could be related to smaller body size in Asian patients. The risk of major bleeding seems to be higher in Asian patients medicated with VKAs than in non‐Asian patients.^16^ In one study, lower NOAC doses were frequently used in Asian patients in routine daily practice. However, unjustified underdosing of apixaban was associated with a less apparent clinical benefit over warfarin in patients.^17^

The data from baseline Phase II of GLORIA‐AF showed that considerable numbers of patients were not treated with OAC, especially in Asia and North America.^18^ Our observations are concordant with other studies in the United States, Denmark, Australia, and Korea.^15^, ^19^, ^20^, ^21^ However, data from the United States (from 2008 to 2014) indicate no increase in OAC prescriptions overall due to an increase in NOAC uptake being offset by a decrease in VKA use.^22^

In our analysis, the prescription of NOAC for stroke prevention has been increasingly associated with individual patient stroke risk as recommended by the European Society of Cardiology guidelines.^23^ The proportion of patients on OAC increased with CHA_2_DS_2_‐VASc score, as described in other datasets.^24^

Increased prescription of NOAC over the 4 years of enrollment in GLORIA‐AF is consistent with other reports.^15^, ^20^, ^25^ The proportion of patients with moderate‐to‐high risk of stroke who are not prescribed OAC has declined continuously. Increased awareness of physicians and patients, improved implementation of guidelines, and educational programs might have resulted in greater NOAC prescription.^26^ Indeed, noticeable differences in patients' baseline characteristics between consecutive years of enrolment are evident. The use of NOAC has also been increasing among patients with higher bleeding risk.

This study has important practical implications and may help in identifying the “action points” needed to improve stroke prevention in AF patients in “routine” clinical practice. Our observations also reflect the evolution of international guidelines on the management of AF in clinical practice.^27^

Numerous registries have reported data on prescription patterns of antithrombotics for stroke prevention in AF, but comparison across registries appears to be challenging for a variety of reasons. The GLORIA‐AF registry's specific design facilitated a description of how the OAC prescription patterns changed across participating countries after NOAC approval. In contrast, in the EURObservational Research Programme (EORP) and the GARFIELD‐AF registries, temporal OAC prescription patterns were presented by calendar year, which led to an aggregation of countries with and without NOAC approval particularly in the first years of NOAC availability; ie, while the first NOAC, dabigatran, was approved in the United States in 2011, the first approval in Italy only occurred in 2013; therefore, country composition as well as the amount patients by country had an impact on the observed treatment patterns. aggregated.^7^, ^25^

In our study, that only started with NOACs availability, the proportion of patients prescribed NOAC increased within a period of between 1 and 4 years, while the proportions of VKA and no‐OAC prescriptions decreased. A pattern similar to that in our study has also been seen in other studies,^25^ with a decline in the use of VKA as well as antiplatelets, and a rise in the use of NOAC. In the EORP‐AF registry ^7^, most of patients who were medicated with VKA or NOAC at the baseline and at 1‐year follow‐up were still anticoagulated with the same OAC at 2‐year follow‐up.

In this study, patients with a high HAS‐BLED score had a generally increasing proportion of OAC prescriptions between the first and fourth year of enrollment. The percentage of patients with no OAC among patients with HAS‐BLED score ≥3 was relatively stable (approximately one third of the patients) over the same period. A high percentage of patients with HAS‐BLED score ≥3 had no OAC prescription, possibly reflecting a lack of concordance between empirical bleeding scores and physician assessment of bleeding risk in AF.^28^ Importantly, there appears to be a need to emphasize that AF patients with a high risk of bleeding should continue taking OAC with close monitoring and frequent visits and individual reassessment of thromboembolic and bleeding risks.^23^, ^29^, ^30^ In the mobile atrial fibrillation application (mAFA‐II) randomized trial, proactive use of HAS‐BLED for dynamic bleeding risk assessment was associated with lower bleeding rates and an increase in OAC use.^31^

Furthermore, year of enrolment, type of site, region, type and categorization of AF, and stroke and bleeding risks are associated with NOAC prescription in the combined Phase II and III data in our analysis. A similar pattern was found in another report where persistent or permanent AF was inversely associated with NOAC prescription.^13^ Also, the year of enrollment was associated with NOAC prescription.

### 4.1 Limitations

These findings may not generalize to the entire global nonvalvular AF patient population or even to the patient population of the participating countries, because the study is restricted to patients with a CHA_2_DS_2_‐VASc score ≥1. Furthermore, this analysis represents only a snapshot of the prescribing practice in the course of treatment and does not take into account treatment continuation, switching or adherence. These issues were addressed in other reports from GLORIA‐AF. Data on the reasons for OAC non‐prescription were not collected.

## 5 CONCLUSIONS

In this global registry of prospectively enrolled AF patients, NOACs have been more commonly prescribed than VKA. During 4 years after approval of the first NOAC for stroke prevention in AF, NOAC use increased over time, while VKA use decreased across all regions.

## CONFLICT OF INTEREST

Dr Kozieł and Professor Rothman declare they have no conflict of interest. Dr Bayer, Gurusamy, and Dr Teutsch are employees of Boehringer Ingelheim. Dr Lu was employee of Boehringer Ingelheim at time of manuscript writing. Dr Diener has received honoraria for participation in clinical trials, contribution to advisory boards or oral presentations from: Abbott, Bayer Vital, Bristol‐Myers Squibb (BMS), Boehringer Ingelheim, Daiichi‐Sankyo, Medtronic, Pfizer, Portola, Sanofi‐Aventis, and WebMD Global. Financial support for research projects was provided by Boehringer Ingelheim. Dr Diener chairs the Treatment Guidelines Committee of the German Society of Neurology and contributed to the EHRA and ESC guidelines for the treatment of AF. Professor Halperin has engaged in consulting activities with Boehringer Ingelheim, for advisory activities involving anticoagulants, and he is a member of the Executive Steering Committee of the GLORIA‐AF Registry. Professor Ma has received honoraria for lectures from AstraZeneca, Bayer HealthCare, Boehringer Ingelheim, BMS, Johnson & Johnson, and Pfizer. Professor Huisman reports grants from ZonMW Dutch Healthcare Fund, grants and personal fees from Boehringer Ingelheim, Pfizer‐BMS, Bayer HealthCare, Aspen, Daiichi‐Sankyo, outside the submitted work. Professor Lip: Consultant and speaker for BMS/Pfizer, Boehringer Ingelheim and Daiichi‐Sankyo. No fees are received personally.

## Supporting information

Table S1

Table S2

Table S3

Table S4
