# Supplementary material for: Changes in anticoagulant prescription patterns over time for patients with atrial fibrillation around the world
Source: J Arrhythm. 2021 Jul 10;37(4):990–1006. doi: 10.1002/joa3.12588 (PMC8339088; doi:10.1002/joa3.12588)
Supplement: Supplementary file 1 — Table S1 [file JOA3-37-990-s002.docx]

**Table S1. Prescription of oral antithrombotic treatment over time by CHA2DS2-VASc score by region**

| **Region:Asia** | **Year 1** | | | **Year 2** | | | | **Year 3** | | | | **Year 4** | | |
| --- | --- | --- | --- | --- | --- | --- | --- | --- | --- | --- | --- | --- | --- | --- |
|  | **CHA2DS2 -VASc score = 1** | **CHA2DS2 -VASc score ≥ 2** | **Overall** | **CHA2DS2 -VASc score = 1** | **CHA2DS2 -VASc score ≥ 2** | **Overall** | **CHA2DS2 -VASc score = 1** | | **CHA2DS2 -VASc score ≥ 2** | **Overall** | **CHA2DS2 -VASc score = 1** | | **CHA2DS2 -VASc score ≥ 2** | **Overall** |
| Number of pts | 477 (100.0) | 1807 (100.0) | 2284 (100.0) | 257 (100.0) | 1079 (100.0) | 1336 (100.0) | 300 (100.0) | | 1096 (100.0) | 1396 | 220 (100.0) | | 717 (100.0) | 937 (100.0) |
| NOAC, n (%) | 78 (16.4) | 588 (32.5) | 666 (29.2) | 102 (39.7) | 517 (47.9) | 619 (46.3) | 88 (29.3) | | 439 (40.1) | 527 (37.8) | 89 (40.5) | | 481 (67.1) | 570 (60.8) |
| On NOACs standard dose, n (%) | 78 (16.4) | 588 (32.5) | 666 (29.2) | 102 (39.7) | 517 (47.9) | 619 (46.3) | 88 (29.3) | | 439 (40.1) | 527 (37.8) | 89 (40.5) | | 481 (67.1) | 570 (60.8) |
| Yes | 35 ( 7.3) | 179 ( 9.9) | 214 ( 9.4) | 47 (18.3) | 192 (17.8) | 239 (17.9) | 37 (12.3) | | 167 (15.2) | 204 (14.6) | 38 (17.3) | | 186 (25.9) | 224 (23.9) |
| No | 43 ( 9.0) | 409 (22.6) | 452 (19.8) | 55 (21.4) | 325 (30.1) | 380 (28.4) | 51 (17.0) | | 272 (24.8) | 323 (23.1) | 51 (23.2) | | 295 (41.1) | 346 (36.9) |
| On NOAC reduced dose, n (%) | 78 (16.4) | 588 (32.5) | 666 (29.2) | 102 (39.7) | 517 (47.9) | 619 (46.3) | 88 (29.3) | | 439 (40.1) | 527 (37.8) | 89 (40.5) | | 481 (67.1) | 570 (60.8) |
| Yes | 43 ( 9.0) | 409 (22.6) | 452 (19.8) | 55 (21.4) | 325 (30.1) | 380 (28.4) | 51 (17.0) | | 272 (24.8) | 323 (23.1) | 51 (23.2) | | 295 (41.1) | 346 (36.9) |
| No | 35 ( 7.3) | 179 ( 9.9) | 214 ( 9.4) | 47 (18.3) | 192 (17.8) | 239 (17.9) | 37 (12.3) | | 167 (15.2) | 204 (14.6) | 38 (17.3) | | 186 (25.9) | 224 (23.9) |
| VKA, n (%) | 136 (28.5) | 458 (25.3) | 594 (26.0) | 48 (18.7) | 218 (20.2) | 266 (19.9) | 64 (21.3) | | 266 (24.3) | 330 (23.6) | 36 (16.4) | | 56 ( 7.8) | 92 ( 9.8) |
| No OAC, n (%) | 263 (55.1) | 761 (42.1) | 1024 (44.8) | 107 (41.6) | 344 (31.9) | 451 (33.8) | 148 (49.3) | | 391 (35.7) | 539 (38.6) | 95 (43.2) | | 180 (25.1) | 275 (29.3) |
| ASA, n (%) | 125 (26.2) | 397 (22.0) | 522 (22.9) | 60 (23.3) | 191 (17.7) | 251 (18.8) | 81 (27.0) | | 232 (21.2) | 313 (22.4) | 50 (22.7) | | 100 (13.9) | 150 (16.0) |
| Antiplts other than ASA, n (%) | 6 ( 1.3) | 28 ( 1.5) | 34 ( 1.5) | 3 ( 1.2) | 19 ( 1.8) | 22 ( 1.6) | 5 ( 1.7) | | 30 ( 2.7) | 35 ( 2.5) | 4 ( 1.8) | | 12 ( 1.7) | 16 ( 1.7) |
| None, n (%) | 132 (27.7) | 336 (18.6) | 468 (20.5) | 44 (17.1) | 134 (12.4) | 178 (13.3) | 62 (20.7) | | 129 (11.8) | 191 (13.7) | 41 (18.6) | | 68 ( 9.5) | 109 (11.6) |
| **Region: Europe** |  |  |  |  |  |  |  | |  |  |  | |  |  |
| Number of patients | 556 (100.0) | 4310 (100.0) | 4866 (100.0) | 491 (100.0) | 3599 (100.0) | 4090 (100.0) | 378 (100.0) | | 2533 (100.0) | 2911 | 204 (100.0) | | 1550 (100.0) | 1754 (100.0) |
| NOAC [N(%)] | 293 (52.7) | 2305 (53.5) | 2598 (53.4) | 252 (51.3) | 2056 (57.1) | 2308 (56.4) | 238 (63.0) | | 1661 (65.6) | 1899 (65.2) | 156 (76.5) | | 1174 (75.7) | 1330 (75.8) |
| On NOACs standard dose [N(%)] | 293 (52.7) | 2305 (53.5) | 2598 (53.4) | 252 (51.3) | 2056 (57.1) | 2308 (56.4) | 238 (63.0) | | 1661 (65.6) | 1899 (65.2) | 156 (76.5) | | 1174 (75.7) | 1330 (75.8) |
| Yes | 248 (44.6) | 1275 (29.6) | 1523 (31.3) | 223 (45.4) | 1291 (35.9) | 1514 (37.0) | 214 (56.6) | | 1159 (45.8) | 1373 (47.2) | 148 (72.5) | | 868 (56.0) | 1016 (57.9) |
| No | 45 ( 8.1) | 1030 (23.9) | 1075 (22.1) | 29 ( 5.9) | 765 (21.3) | 794 (19.4) | 24 ( 6.3) | | 502 (19.8) | 526 (18.1) | 8 ( 3.9) | | 306 (19.7) | 314 (17.9) |
| On NOACs reduced dose [N(%)] | 293 (52.7) | 2305 (53.5) | 2598 (53.4) | 252 (51.3) | 2056 (57.1) | 2308 (56.4) | 238 (63.0) | | 1661 (65.6) | 1899 (65.2) | 156 (76.5) | | 1174 (75.7) | 1330 (75.8) |
| Yes | 45 ( 8.1) | 1030 (23.9) | 1075 (22.1) | 29 ( 5.9) | 765 (21.3) | 794 (19.4) | 24 ( 6.3) | | 502 (19.8) | 526 (18.1) | 8 ( 3.9) | | 306 (19.7) | 314 (17.9) |
| No | 248 (44.6) | 1275 (29.6) | 1523 (31.3) | 223 (45.4) | 1291 (35.9) | 1514 (37.0) | 214 (56.6) | | 1159 (45.8) | 1373 (47.2) | 148 (72.5) | | 868 (56.0) | 1016 (57.9) |
| VKA [N(%)] | 160 (28.8) | 1568 (36.4) | 1728 (35.5) | 155 (31.6) | 1212 (33.7) | 1367 (33.4) | 74 (19.6) | | 667 (26.3) | 741 (25.5) | 17 ( 8.3) | | 278 (17.9) | 295 (16.8) |
| No OAC [N(%)] | 103 (18.5) | 437 (10.1) | 540 (11.1) | 84 (17.1) | 331 ( 9.2) | 415 (10.1) | 66 (17.5) | | 205 ( 8.1) | 271 ( 9.3) | 31 (15.2) | | 98 ( 6.3) | 129 ( 7.4) |
| ASA [N(%)] | 47 ( 8.5) | 233 ( 5.4) | 280 ( 5.8) | 37 ( 7.5) | 191 ( 5.3) | 228 ( 5.6) | 24 ( 6.3) | | 91 ( 3.6) | 115 ( 4.0) | 10 ( 4.9) | | 56 ( 3.6) | 66 ( 3.8) |
| Antiplts other than ASA [N(%)] | 2 ( 0.4) | 41 ( 1.0) | 43 ( 0.9) | 2 ( 0.4) | 34 ( 0.9) | 36 ( 0.9) | 2 ( 0.5) | | 20 ( 0.8) | 22 ( 0.8) | 0 ( 0.0) | | 4 ( 0.3) | 4 ( 0.2) |
| None [N(%)] | 54 ( 9.7) | 163 ( 3.8) | 217 ( 4.5) | 45 ( 9.2) | 106 ( 2.9) | 151 ( 3.7) | 40 (10.6) | | 94 ( 3.7) | 134 ( 4.6) | 21 (10.3) | | 38 ( 2.5) | 59 ( 3.4) |
| **Region: North America** |  |  |  |  |  |  |  | |  |  |  | |  |  |
| Number of patients | 202 (100.0) | 1256 (100.0) | 1458 (100.0) | 281 (100.0) | 1764 (100.0) | 2045 (100.0) | 220 (100.0) | | 1373 (100.0) | 1593 | 181 (100.0) | | 1077 (100.0) | 1258 (100.0) |
| NOAC [N(%)] | 91 (45.0) | 623 (49.6) | 714 (49.0) | 157 (55.9) | 1058 (60.0) | 1215 (59.4) | 134 (60.9) | | 959 (69.8) | 1093 (68.6) | 118 (65.2) | | 812 (75.4) | 930 (73.9) |
| On NOACs standard dose [N(%)] | 91 (45.0) | 623 (49.6) | 714 (49.0) | 157 (55.9) | 1058 (60.0) | 1215 (59.4) | 134 (60.9) | | 959 (69.8) | 1093 (68.6) | 118 (65.2) | | 812 (75.4) | 930 (73.9) |
| Yes | 88 (43.6) | 511 (40.7) | 599 (41.1) | 147 (52.3) | 886 (50.2) | 1033 (50.5) | 133 (60.5) | | 810 (59.0) | 943 (59.2) | 110 (60.8) | | 662 (61.5) | 772 (61.4) |
| No | 3 ( 1.5) | 112 ( 8.9) | 115 ( 7.9) | 10 ( 3.6) | 172 ( 9.8) | 182 ( 8.9) | 1 ( 0.5) | | 149 (10.9) | 150 ( 9.4) | 8 ( 4.4) | | 150 (13.9) | 158 (12.6) |
| On NOACs reduced dose [N(%)] | 91 (45.0) | 623 (49.6) | 714 (49.0) | 157 (55.9) | 1058 (60.0) | 1215 (59.4) | 134 (60.9) | | 959 (69.8) | 1093 (68.6) | 118 (65.2) | | 812 (75.4) | 930 (73.9) |
| Yes | 3 ( 1.5) | 112 ( 8.9) | 115 ( 7.9) | 10 ( 3.6) | 172 ( 9.8) | 182 ( 8.9) | 1 ( 0.5) | | 149 (10.9) | 150 ( 9.4) | 8 ( 4.4) | | 150 (13.9) | 158 (12.6) |
| No | 88 (43.6) | 511 (40.7) | 599 (41.1) | 147 (52.3) | 886 (50.2) | 1033 (50.5) | 133 (60.5) | | 810 (59.0) | 943 (59.2) | 110 (60.8) | | 662 (61.5) | 772 (61.4) |
| VKA [N(%)] | 38 (18.8) | 384 (30.6) | 422 (28.9) | 30 (10.7) | 412 (23.4) | 442 (21.6) | 21 ( 9.5) | | 195 (14.2) | 216 (13.6) | 8 ( 4.4) | | 144 (13.4) | 152 (12.1) |
| No OAC [N(%)] | 73 (36.1) | 249 (19.8) | 322 (22.1) | 94 (33.5) | 294 (16.7) | 388 (19.0) | 65 (29.5) | | 219 (16.0) | 284 (17.8) | 55 (30.4) | | 121 (11.2) | 176 (14.0) |
| ASA [N(%)] | 57 (28.2) | 143 (11.4) | 200 (13.7) | 78 (27.8) | 184 (10.4) | 262 (12.8) | 52 (23.6) | | 148 (10.8) | 200 (12.6) | 43 (23.8) | | 91 ( 8.4) | 134 (10.7) |
| Antiplts other than ASA [N(%)] | 0 ( 0.0) | 4 ( 0.3) | 4 ( 0.3) | 0 ( 0.0) | 21 ( 1.2) | 21 ( 1.0) | 0 ( 0.0) | | 5 ( 0.4) | 5 ( 0.3) | 0 ( 0.0) | | 4 ( 0.4) | 4 ( 0.3) |
| None [N(%)] | 16 ( 7.9) | 102 ( 8.1) | 118 ( 8.1) | 16 ( 5.7) | 89 ( 5.0) | 105 ( 5.1) | 13 ( 5.9) | | 66 ( 4.8) | 79 ( 5.0) | 12 ( 6.6) | | 26 ( 2.4) | 38 ( 3.0) |
| **Region: Latin America** |  |  |  |  |  |  |  | |  |  |  | |  |  |
| Number of patients | 39 (100.0) | 322 (100.0) | 361 (100.0) | 59 (100.0) | 361 (100.0) | 420 (100.0) | 40 (100.0) | | 244 (100.0) | 284 | 50 (100.0) | | 389 (100.0) | 439 (100.0) |
| NOAC [N(%)] | 20 (51.3) | 181 (56.2) | 201 (55.7) | 27 (45.8) | 192 (53.2) | 219 (52.1) | 21 (52.5) | | 143 (58.6) | 164 (57.7) | 38 (76.0) | | 274 (70.4) | 312 (71.1) |
| On NOACs standard dose [N(%)] | 20 (51.3) | 181 (56.2) | 201 (55.7) | 27 (45.8) | 192 (53.2) | 219 (52.1) | 21 (52.5) | | 143 (58.6) | 164 (57.7) | 38 (76.0) | | 274 (70.4) | 312 (71.1) |
| Yes | 18 (46.2) | 104 (32.3) | 122 (33.8) | 22 (37.3) | 90 (24.9) | 112 (26.7) | 13 (32.5) | | 70 (28.7) | 83 (29.2) | 29 (58.0) | | 113 (29.0) | 142 (32.3) |
| No | 2 ( 5.1) | 77 (23.9) | 79 (21.9) | 5 ( 8.5) | 102 (28.3) | 107 (25.5) | 8 (20.0) | | 73 (29.9) | 81 (28.5) | 9 (18.0) | | 161 (41.4) | 170 (38.7) |
| On NOACs reduced dose [N(%)] | 20 (51.3) | 181 (56.2) | 201 (55.7) | 27 (45.8) | 192 (53.2) | 219 (52.1) | 21 (52.5) | | 143 (58.6) | 164 (57.7) | 38 (76.0) | | 274 (70.4) | 312 (71.1) |
| Yes | 2 ( 5.1) | 77 (23.9) | 79 (21.9) | 5 ( 8.5) | 102 (28.3) | 107 (25.5) | 8 (20.0) | | 73 (29.9) | 81 (28.5) | 9 (18.0) | | 161 (41.4) | 170 (38.7) |
| No | 18 (46.2) | 104 (32.3) | 122 (33.8) | 22 (37.3) | 90 (24.9) | 112 (26.7) | 13 (32.5) | | 70 (28.7) | 83 (29.2) | 29 (58.0) | | 113 (29.0) | 142 (32.3) |
| VKA [N(%)] | 13 (33.3) | 104 (32.3) | 117 (32.4) | 19 (32.2) | 118 (32.7) | 137 (32.6) | 9 (22.5) | | 64 (26.2) | 73 (25.7) | 11 (22.0) | | 67 (17.2) | 78 (17.8) |
| No OAC [N(%)] | 6 (15.4) | 37 (11.5) | 43 (11.9) | 13 (22.0) | 51 (14.1) | 64 (15.2) | 10 (25.0) | | 37 (15.2) | 47 (16.5) | 1 ( 2.0) | | 48 (12.3) | 49 (11.2) |
| ASA [N(%)] | 3 ( 7.7) | 21 ( 6.5) | 24 ( 6.6) | 9 (15.3) | 37 (10.2) | 46 (11.0) | 8 (20.0) | | 25 (10.2) | 33 (11.6) | 0 ( 0.0) | | 31 ( 8.0) | 31 ( 7.1) |
| Antiplts other than ASA [N(%)] | 0 ( 0.0) | 3 ( 0.9) | 3 ( 0.8) | 1 ( 1.7) | 3 ( 0.8) | 4 ( 1.0) | 0 ( 0.0) | | 3 ( 1.2) | 3 ( 1.1) | 0 ( 0.0) | | 3 ( 0.8) | 3 ( 0.7) |
| None [N(%)] | 3 ( 7.7) | 13 ( 4.0) | 16 ( 4.4) | 3 ( 5.1) | 11 ( 3.0) | 14 ( 3.3) | 2 ( 5.0) | | 9 ( 3.7) | 11 ( 3.9) | 1 ( 2.0) | | 14 ( 3.6) | 15 ( 3.4) |

ASA, acetylsalycylic acid, CHA_2_DS_2_-VASc, congestive heart failure, hypertension, age ≥75 years, diabetes, stroke/transient ischaemic attack/systemic embolism, vascular disease, age 65-74 years, sex category (female), NOAC, non-vitamin K antagonist oral anticoagulants, VKA, vitamin K antagonists.

Standard dose: Dabigatran 150 mg BID, Rivaroxaban 20 mg QD, Apixaban 5 mg BID, Edoxaban 60 mg QD. The other doses are reduced.
