# Supplementary material for: Changes in anticoagulant prescription patterns over time for patients with atrial fibrillation around the world
Source: J Arrhythm. 2021 Jul 10;37(4):990–1006. doi: 10.1002/joa3.12588 (PMC8339088; doi:10.1002/joa3.12588)
Supplement: Supplementary file 2 — Table S2 [file JOA3-37-990-s001.docx]

**Table S2. Interval change in oral anticoagulants by CHA_2_DS_2_-VASc and region**

|  | **NOAC** | | **VKA** | |
| --- | --- | --- | --- | --- |
| Region | CHA_2_DS_2_-VASc score ≥2 | CHA_2_DS_2_-VASc score = 1 | CHA_2_DS_2_-VASc score ≥2 | CHA_2_DS_2_-VASc score = 1 |
| Asia | 34.6% | 24.1% | -17.5% | -12.2% |
| Europe | 22.2% | 23.8% | -18.5% | -20.5% |
| North America | 25.8% | 20.2% | -17.2% | -14.4% |
| Latin America | 14.2% | 24.7% | -15.3% | -11.3% |

CHA_2_DS_2_-VASc, congestive heart failure, hypertension, age ≥75 years, diabetes, stroke/transient ischaemic attack/systemic embolism, vascular disease, age 65-74 years, sex category (female), NOAC, non-vitamin K antagonist oral anticoagulants, VKA, vitamin K antagonists.
