# Supplementary material for: Changes in anticoagulant prescription patterns over time for patients with atrial fibrillation around the world
Source: J Arrhythm. 2021 Jul 10;37(4):990–1006. doi: 10.1002/joa3.12588 (PMC8339088; doi:10.1002/joa3.12588)
Supplement: Supplementary file 3 — Table S3 [file JOA3-37-990-s003.docx]

**Table S3. Prescription of oral antithrombotic treatment over time by HAS-BLED score by region**

| **Region:Asia** | **Year 1** | | | **Year 2** | | | **Year 3** | | | **Year 4** | | |
| --- | --- | --- | --- | --- | --- | --- | --- | --- | --- | --- | --- | --- |
|  | **HAS-BLED <3** | **HAS-BLED ≥3** | **Overall** | **HAS-BLED <3** | **HAS-BLED ≥3** | **Overall** | **HAS-BLED <3** | **HAS-BLED ≥3** | **Overall** | **HAS-BLED <3** | **HAS-BLED ≥3** | **Overall** |
| Number of pts | 1747 (100.0) | 253 (100.0) | 2000 (100.0) | 1004 (100.0) | 148 (100.0) | 1152 (100.0) | 1160 (100.0) | 153 (100.0) | 1313 | 751 (100.0) | 75 (100.0) | 826 (100.0) |
| NOAC, n (%) | 569 (32.6) | 47 (18.6) | 616 (30.8) | 448 (44.6) | 48 (32.4) | 496 (43.1) | 473 (40.8) | 27 (17.6) | 500 (38.1) | 473 (63.0) | 17 (22.7) | 490 (59.3) |
| On NOACs standard dose, n (%) | 569 (32.6) | 47 (18.6) | 616 (30.8) | 448 (44.6) | 48 (32.4) | 496 (43.1) | 473 (40.8) | 27 (17.6) | 500 (38.1) | 473 (63.0) | 17 (22.7) | 490 (59.3) |
| Yes | 186 (10.6) | 12 ( 4.7) | 198 ( 9.9) | 174 (17.3) | 14 ( 9.5) | 188 (16.3) | 186 (16.0) | 7 ( 4.6) | 193 (14.7) | 188 (25.0) | 3 ( 4.0) | 191 (23.1) |
| No | 383 (21.9) | 35 (13.8) | 418 (20.9) | 274 (27.3) | 34 (23.0) | 308 (26.7) | 287 (24.7) | 20 (13.1) | 307 (23.4) | 285 (37.9) | 14 (18.7) | 299 (36.2) |
| On NOAC reduced dose, n (%) | 569 (32.6) | 47 (18.6) | 616 (30.8) | 448 (44.6) | 48 (32.4) | 496 (43.1) | 473 (40.8) | 27 (17.6) | 500 (38.1) | 473 (63.0) | 17 (22.7) | 490 (59.3) |
| Yes | 383 (21.9) | 35 (13.8) | 418 (20.9) | 274 (27.3) | 34 (23.0) | 308 (26.7) | 287 (24.7) | 20 (13.1) | 307 (23.4) | 285 (37.9) | 14 (18.7) | 299 (36.2) |
| No | 186 (10.6) | 12 ( 4.7) | 198 ( 9.9) | 174 (17.3) | 14 ( 9.5) | 188 (16.3) | 186 (16.0) | 7 ( 4.6) | 193 (14.7) | 188 (25.0) | 3 ( 4.0) | 191 (23.1) |
| VKA, n (%) | 451 (25.8) | 48 (19.0) | 499 (25.0) | 225 (22.4) | 15 (10.1) | 240 (20.8) | 293 (25.3) | 17 (11.1) | 310 (23.6) | 79 (10.5) | 7 ( 9.3) | 86 (10.4) |
| No OAC, n (%) | 727 (41.6) | 158 (62.5) | 885 (44.3) | 331 (33.0) | 85 (57.4) | 416 (36.1) | 394 (34.0) | 109 (71.2) | 503 (38.3) | 199 (26.5) | 51 (68.0) | 250 (30.3) |
| ASA, n (%) | 336 (19.2) | 119 (47.0) | 455 (22.8) | 165 (16.4) | 68 (45.9) | 233 (20.2) | 207 (17.8) | 85 (55.6) | 292 (22.2) | 99 (13.2) | 37 (49.3) | 136 (16.5) |
| Antiplts other than ASA, n (%) | 15 ( 0.9) | 14 ( 5.5) | 29 ( 1.5) | 14 ( 1.4) | 8 ( 5.4) | 22 ( 1.9) | 24 ( 2.1) | 10 ( 6.5) | 34 ( 2.6) | 7 ( 0.9) | 7 ( 9.3) | 14 ( 1.7) |
| None, n (%) | 376 (21.5) | 25 ( 9.9) | 401 (20.1) | 152 (15.1) | 9 ( 6.1) | 161 (14.0) | 163 (14.1) | 14 ( 9.2) | 177 (13.5) | 93 (12.4) | 7 ( 9.3) | 100 (12.1) |
| **Region: Europe** |  |  |  |  |  |  |  |  |  |  |  |  |
| Number of patients | 3837 (100.0) | 401 (100.0) | 4238 (100.0) | 3226 (100.0) | 356 (100.0) | 3582 (100.0) | 2357 (100.0) | 211 (100.0) | 2568 | 1404 (100.0) | 116 (100.0) | 1520 (100.0) |
| NOAC [N(%)] | 2092 (54.5) | 152 (37.9) | 2244 (52.9) | 1840 (57.0) | 172 (48.3) | 2012 (56.2) | 1549 (65.7) | 132 (62.6) | 1681 (65.5) | 1089 (77.6) | 68 (58.6) | 1157 (76.1) |
| On NOACs standard dose [N(%)] | 2092 (54.5) | 152 (37.9) | 2244 (52.9) | 1840 (57.0) | 172 (48.3) | 2012 (56.2) | 1549 (65.7) | 132 (62.6) | 1681 (65.5) | 1089 (77.6) | 68 (58.6) | 1157 (76.1) |
| Yes | 1238 (32.3) | 66 (16.5) | 1304 (30.8) | 1203 (37.3) | 86 (24.2) | 1289 (36.0) | 1137 (48.2) | 68 (32.2) | 1205 (46.9) | 837 (59.6) | 43 (37.1) | 880 (57.9) |
| No | 854 (22.3) | 86 (21.4) | 940 (22.2) | 637 (19.7) | 86 (24.2) | 723 (20.2) | 412 (17.5) | 64 (30.3) | 476 (18.5) | 252 (17.9) | 25 (21.6) | 277 (18.2) |
| On NOACs reduced dose [N(%)] | 2092 (54.5) | 152 (37.9) | 2244 (52.9) | 1840 (57.0) | 172 (48.3) | 2012 (56.2) | 1549 (65.7) | 132 (62.6) | 1681 (65.5) | 1089 (77.6) | 68 (58.6) | 1157 (76.1) |
| Yes | 854 (22.3) | 86 (21.4) | 940 (22.2) | 637 (19.7) | 86 (24.2) | 723 (20.2) | 412 (17.5) | 64 (30.3) | 476 (18.5) | 252 (17.9) | 25 (21.6) | 277 (18.2) |
| No | 1238 (32.3) | 66 (16.5) | 1304 (30.8) | 1203 (37.3) | 86 (24.2) | 1289 (36.0) | 1137 (48.2) | 68 (32.2) | 1205 (46.9) | 837 (59.6) | 43 (37.1) | 880 (57.9) |
| VKA [N(%)] | 1386 (36.1) | 138 (34.4) | 1524 (36.0) | 1098 (34.0) | 111 (31.2) | 1209 (33.8) | 610 (25.9) | 35 (16.6) | 645 (25.1) | 240 (17.1) | 17 (14.7) | 257 (16.9) |
| No OAC [N(%)] | 359 ( 9.4) | 111 (27.7) | 470 (11.1) | 288 ( 8.9) | 73 (20.5) | 361 (10.1) | 198 ( 8.4) | 44 (20.9) | 242 ( 9.4) | 75 ( 5.3) | 31 (26.7) | 106 ( 7.0) |
| ASA [N(%)] | 170 ( 4.4) | 78 (19.5) | 248 ( 5.9) | 149 ( 4.6) | 48 (13.5) | 197 ( 5.5) | 78 ( 3.3) | 23 (10.9) | 101 ( 3.9) | 37 ( 2.6) | 21 (18.1) | 58 ( 3.8) |
| Antiplts other than ASA [N(%)] | 17 ( 0.4) | 20 ( 5.0) | 37 ( 0.9) | 15 ( 0.5) | 17 ( 4.8) | 32 ( 0.9) | 9 ( 0.4) | 11 ( 5.2) | 20 ( 0.8) | 2 ( 0.1) | 1 ( 0.9) | 3 ( 0.2) |
| None [N(%)] | 172 ( 4.5) | 13 ( 3.2) | 185 ( 4.4) | 124 ( 3.8) | 8 ( 2.2) | 132 ( 3.7) | 111 ( 4.7) | 10 ( 4.7) | 121 ( 4.7) | 36 ( 2.6) | 9 ( 7.8) | 45 ( 3.0) |
| **Region: North America** |  |  |  |  |  |  |  |  |  |  |  |  |
| Number of patients | 1186 (100.0) | 131 (100.0) | 1317 (100.0) | 1668 (100.0) | 238 (100.0) | 1906 (100.0) | 1342 (100.0) | 168 (100.0) | 1510 | 1054 (100.0) | 130 (100.0) | 1184 (100.0) |
| NOAC [N(%)] | 593 (50.0) | 57 (43.5) | 650 (49.4) | 1021 (61.2) | 120 (50.4) | 1141 (59.9) | 947 (70.6) | 89 (53.0) | 1036 (68.6) | 798 (75.7) | 83 (63.8) | 881 (74.4) |
| On NOACs standard dose [N(%)] | 593 (50.0) | 57 (43.5) | 650 (49.4) | 1021 (61.2) | 120 (50.4) | 1141 (59.9) | 947 (70.6) | 89 (53.0) | 1036 (68.6) | 798 (75.7) | 83 (63.8) | 881 (74.4) |
| Yes | 505 (42.6) | 41 (31.3) | 546 (41.5) | 875 (52.5) | 94 (39.5) | 969 (50.8) | 819 (61.0) | 74 (44.0) | 893 (59.1) | 669 (63.5) | 61 (46.9) | 730 (61.7) |
| No | 88 ( 7.4) | 16 (12.2) | 104 ( 7.9) | 146 ( 8.8) | 26 (10.9) | 172 ( 9.0) | 128 ( 9.5) | 15 ( 8.9) | 143 ( 9.5) | 129 (12.2) | 22 (16.9) | 151 (12.8) |
| On NOACs reduced dose [N(%)] | 593 (50.0) | 57 (43.5) | 650 (49.4) | 1021 (61.2) | 120 (50.4) | 1141 (59.9) | 947 (70.6) | 89 (53.0) | 1036 (68.6) | 798 (75.7) | 83 (63.8) | 881 (74.4) |
| Yes | 88 ( 7.4) | 16 (12.2) | 104 ( 7.9) | 146 ( 8.8) | 26 (10.9) | 172 ( 9.0) | 128 ( 9.5) | 15 ( 8.9) | 143 ( 9.5) | 129 (12.2) | 22 (16.9) | 151 (12.8) |
| No | 505 (42.6) | 41 (31.3) | 546 (41.5) | 875 (52.5) | 94 (39.5) | 969 (50.8) | 819 (61.0) | 74 (44.0) | 893 (59.1) | 669 (63.5) | 61 (46.9) | 730 (61.7) |
| VKA [N(%)] | 340 (28.7) | 32 (24.4) | 372 (28.2) | 355 (21.3) | 52 (21.8) | 407 (21.4) | 173 (12.9) | 29 (17.3) | 202 (13.4) | 123 (11.7) | 19 (14.6) | 142 (12.0) |
| No OAC [N(%)] | 253 (21.3) | 42 (32.1) | 295 (22.4) | 292 (17.5) | 66 (27.7) | 358 (18.8) | 222 (16.5) | 50 (29.8) | 272 (18.0) | 133 (12.6) | 28 (21.5) | 161 (13.6) |
| ASA [N(%)] | 146 (12.3) | 36 (27.5) | 182 (13.8) | 193 (11.6) | 49 (20.6) | 242 (12.7) | 149 (11.1) | 43 (25.6) | 192 (12.7) | 98 ( 9.3) | 25 (19.2) | 123 (10.4) |
| Antiplts other than ASA [N(%)] | 1 ( 0.1) | 3 ( 2.3) | 4 ( 0.3) | 10 ( 0.6) | 8 ( 3.4) | 18 ( 0.9) | 3 ( 0.2) | 1 ( 0.6) | 4 ( 0.3) | 1 ( 0.1) | 2 ( 1.5) | 3 ( 0.3) |
| None [N(%)] | 106 ( 8.9) | 3 ( 2.3) | 109 ( 8.3) | 89 ( 5.3) | 9 ( 3.8) | 98 ( 5.1) | 70 ( 5.2) | 6 ( 3.6) | 76 ( 5.0) | 34 ( 3.2) | 1 ( 0.8) | 35 ( 3.0) |
| **Region: Latin America** |  |  |  |  |  |  |  |  |  |  |  |  |
| Number of patients | 299 (100.0) | 16 (100.0) | 315 (100.0) | 355 (100.0) | 28 (100.0) | 383 (100.0) | 233 (100.0) | 18 (100.0) | 251 | 410 (100.0) | 20 (100.0) | 430 (100.0) |
| NOAC [N(%)] | 172 (57.5) | 6 (37.5) | 178 (56.5) | 194 (54.6) | 9 (32.1) | 203 (53.0) | 136 (58.4) | 11 (61.1) | 147 (58.6) | 294 (71.7) | 12 (60.0) | 306 (71.2) |
| On NOACs standard dose [N(%)] | 172 (57.5) | 6 (37.5) | 178 (56.5) | 194 (54.6) | 9 (32.1) | 203 (53.0) | 136 (58.4) | 11 (61.1) | 147 (58.6) | 294 (71.7) | 12 (60.0) | 306 (71.2) |
| Yes | 104 (34.8) | 3 (18.8) | 107 (34.0) | 98 (27.6) | 5 (17.9) | 103 (26.9) | 66 (28.3) | 4 (22.2) | 70 (27.9) | 136 (33.2) | 4 (20.0) | 140 (32.6) |
| No | 68 (22.7) | 3 (18.8) | 71 (22.5) | 96 (27.0) | 4 (14.3) | 100 (26.1) | 70 (30.0) | 7 (38.9) | 77 (30.7) | 158 (38.5) | 8 (40.0) | 166 (38.6) |
| On NOACs reduced dose [N(%)] | 172 (57.5) | 6 (37.5) | 178 (56.5) | 194 (54.6) | 9 (32.1) | 203 (53.0) | 136 (58.4) | 11 (61.1) | 147 (58.6) | 294 (71.7) | 12 (60.0) | 306 (71.2) |
| Yes | 68 (22.7) | 3 (18.8) | 71 (22.5) | 96 (27.0) | 4 (14.3) | 100 (26.1) | 70 (30.0) | 7 (38.9) | 77 (30.7) | 158 (38.5) | 8 (40.0) | 166 (38.6) |
| No | 104 (34.8) | 3 (18.8) | 107 (34.0) | 98 (27.6) | 5 (17.9) | 103 (26.9) | 66 (28.3) | 4 (22.2) | 70 (27.9) | 136 (33.2) | 4 (20.0) | 140 (32.6) |
| VKA [N(%)] | 92 (30.8) | 5 (31.3) | 97 (30.8) | 116 (32.7) | 8 (28.6) | 124 (32.4) | 61 (26.2) | 6 (33.3) | 67 (26.7) | 74 (18.0) | 2 (10.0) | 76 (17.7) |
| No OAC [N(%)] | 35 (11.7) | 5 (31.3) | 40 (12.7) | 45 (12.7) | 11 (39.3) | 56 (14.6) | 36 (15.5) | 1 ( 5.6) | 37 (14.7) | 42 (10.2) | 6 (30.0) | 48 (11.2) |
| ASA [N(%)] | 18 ( 6.0) | 5 (31.3) | 23 ( 7.3) | 32 ( 9.0) | 10 (35.7) | 42 (11.0) | 23 ( 9.9) | 1 ( 5.6) | 24 ( 9.6) | 25 ( 6.1) | 5 (25.0) | 30 ( 7.0) |
| Antiplts other than ASA [N(%)] | 2 ( 0.7) | 0 ( 0.0) | 2 ( 0.6) | 3 ( 0.8) | 0 ( 0.0) | 3 ( 0.8) | 3 ( 1.3) | 0 ( 0.0) | 3 ( 1.2) | 3 ( 0.7) | 0 ( 0.0) | 3 ( 0.7) |
| None [N(%)] | 15 ( 5.0) | 0 ( 0.0) | 15 ( 4.8) | 10 ( 2.8) | 1 ( 3.6) | 11 ( 2.9) | 10 ( 4.3) | 0 ( 0.0) | 10 ( 4.0) | 14 ( 3.4) | 1 ( 5.0) | 15 ( 3.5) |

ASA, acetylsalycylic acid, HAS-BLED, (hypertension, abnormal renal/liver function, stroke, bleeding history or predisposition, labile International Normalised Ratio (INR), elderly (>65 years), drugs or alcohol concomitantly, NOAC, non-vitamin K antagonist oral anticoagulants, VKA, vitamin K antagonists.

Standard dose: Dabigatran 150 mg BID, Rivaroxaban 20 mg QD, Apixaban 5 mg BID, Edoxaban 60 mg QD. The other doses are reduced.
