# Supplementary material for: Changes in anticoagulant prescription patterns over time for patients with atrial fibrillation around the world
Source: J Arrhythm. 2021 Jul 10;37(4):990–1006. doi: 10.1002/joa3.12588 (PMC8339088; doi:10.1002/joa3.12588)
Supplement: Supplementary file 4 — Table S4 [file JOA3-37-990-s004.docx]

Table S4. Interval change in oral anticoagulants by HAS-BLED and region

|  | **NOAC** | | **VKA** | |
| --- | --- | --- | --- | --- |
| Region | HAS-BLED score ≥3 | HAS-BLED score <3 | HAS-BLED score ≥3 | HAS-BLED score <3 |
| Asia | 4.1% | 30.4% | -9.7% | -12.2% |
| Europe | 20.7% | 23.1% | -18.5% | -15.3% |
| North America | 20.3% | 25.7% | -17.2% | -19.0% |
| Latin America | 22.5% | 14.2% | -21.3% | -17.0% |

HAS-BLED, (hypertension, abnormal renal/liver function, stroke, bleeding history or predisposition, labile International Normalised Ratio (INR), elderly (>65 years), drugs or alcohol concomitantly, NOAC, non-vitamin K antagonist oral anticoagulants, VKA, vitamin K antagonists.
